# Supplementary material for: Determinants of Spike infectivity, processing, and neutralization in SARS-CoV-2 Omicron subvariants BA.1 and BA.2
Source: Cell Host Microbe. 2022 Sep 14;30(9):1255–1268.e5. doi: 10.1016/j.chom.2022.07.006 (PMC9289044; doi:10.1016/j.chom.2022.07.006)
Supplement: Document S1. Figures S1–S3 and Tables S1 and S2 [file mmc1.pdf]

Cell Host & Microbe, Volume *30*

## Supplemental information

# Determinants of Spike infectivity, processing, and neutralization in SARS-CoV-2 Omicron subvariants BA.1 and BA.2

**Chiara Pastorio, Fabian Zech, Sabrina Noettger, Christoph Jung, Timo Jacob, Theo Sanderson, Konstantin M.J. Sparrer, and Frank Kirchhoff**

# SUPPLEMENTAL FIGURES

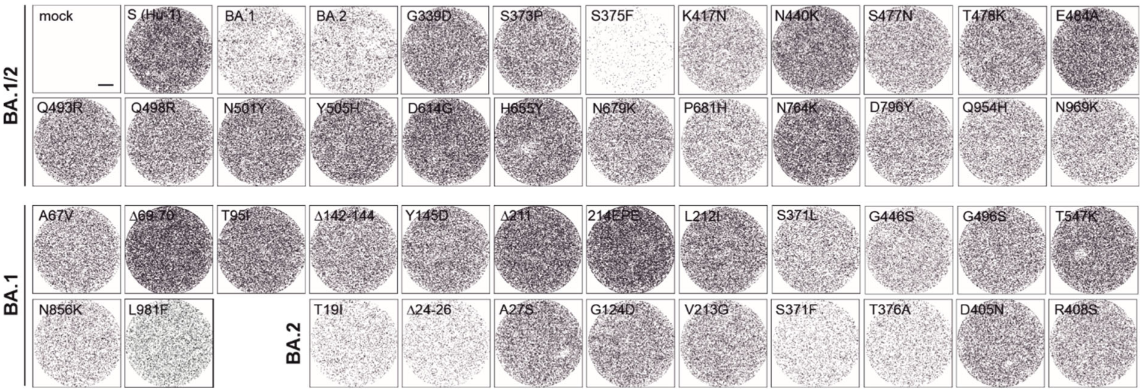


**Figure S1 (related to Figure 2). Infection of CaCo-2 cells by VSVpp containing WT or mutant S proteins.**

Images of CaCo-2 cells transduced with VSVΔG-GFP pseudotyped with the Hu-1 or mutant SARS-CoV-2 S proteins. Successful infection events (GFP+ cells) are displayed as black dots. Scale bar, 1.5 μm.

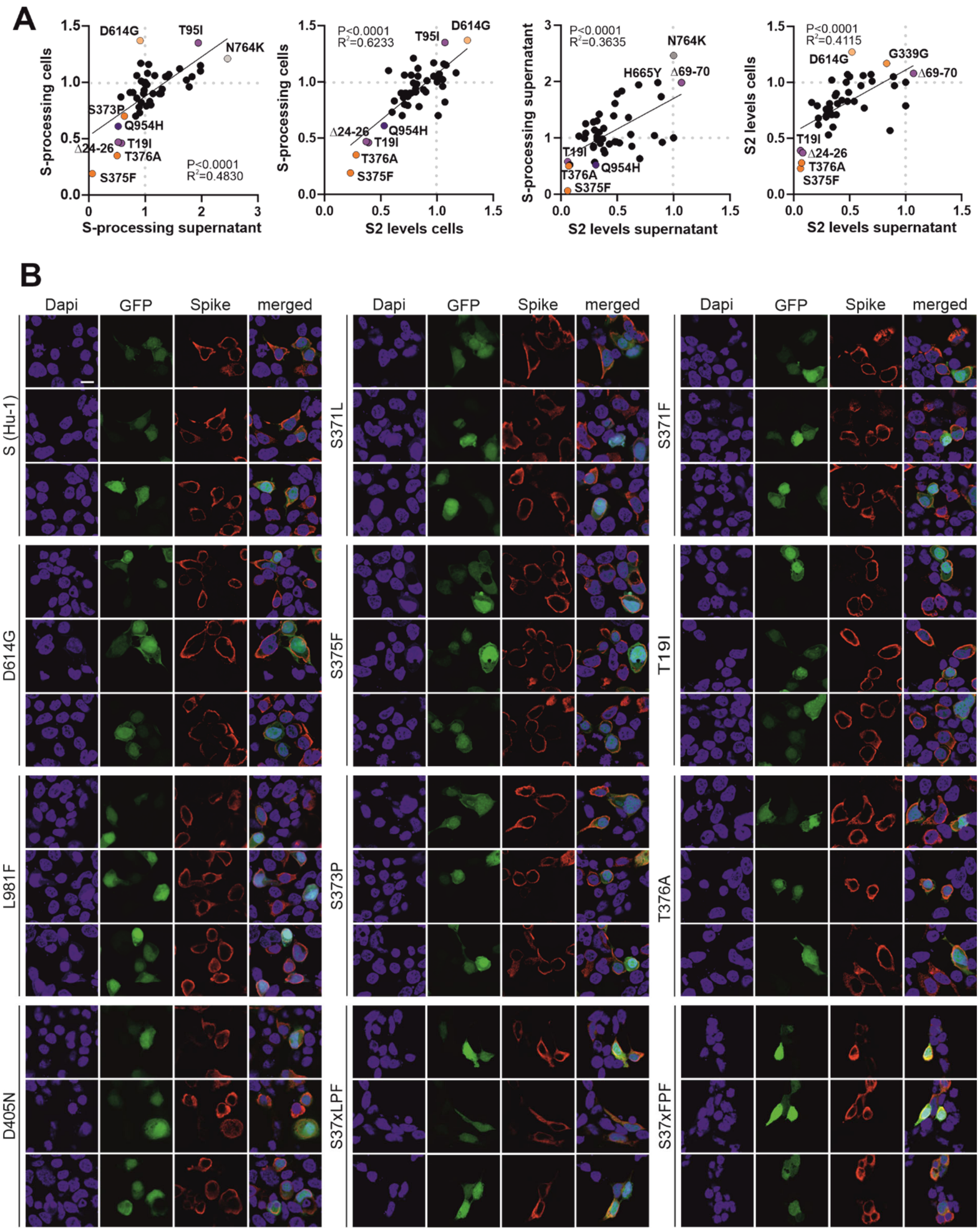


**Figure S2 (related to Figure 3). Correlation analyses and localization of S proteins.**

(A) Correlation of the between the indicated parameters. S2 expression levels and S/S2 processing of mutant S proteins were normalized to the parental Hu-1 S (set to 1).

(B) Immunofluorescence images of HEK293T cells expressing the parental Hu-1 or indicated mutant S proteins. Scale bar, 10 μm.

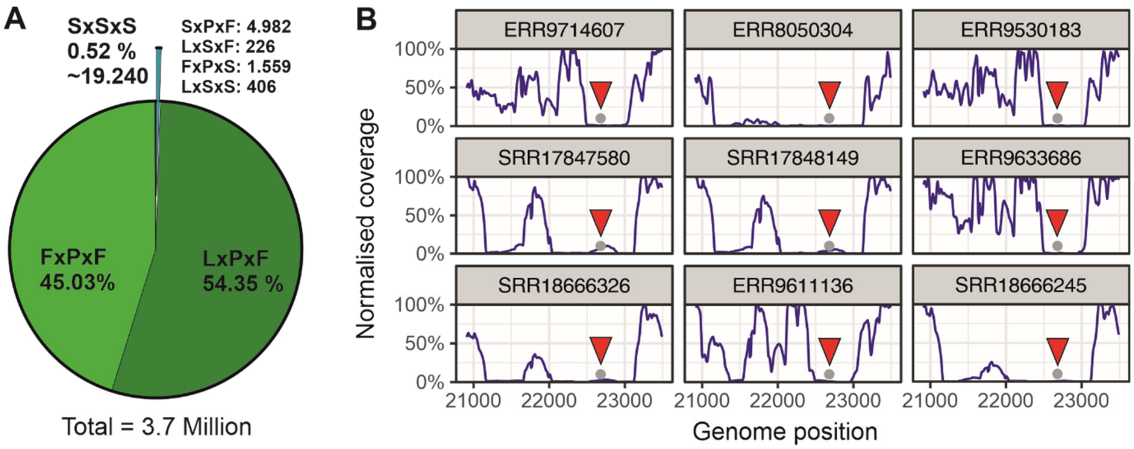


**Figure S3 (related to Figure 4). Apparent changes to serine residues at amino acid positions 371, 373 and 375 of Omicron Spike.** (A) Pie chart depicting the frequency of GSAID deposited sequences with indicated Spike mutations, retrieved from CoVspectrum on 23.06.2022. (B) Normalised coverage plots for randomly selected Omicron genomes with consensus sequences having a "revertant" S:371S show drops of coverage at this position. The red arrow indicates the S:371-5 location.

# SUPPLEMENTAL TABLES

**Table S1 (related to Figure 5).** Impact of mutations in Omicron BA.1 and BA.2 Spike on infection, processing, ACE2 binding, syncytia formation and neutralization.

| **Residue** | **Infection** | **Processing** | | | | **ACE2 interaction** | **Syncytia formation** | **Serum neutralization** |
|---|---|---|---|---|---|---|---|---|
| | | S Sups | S2 Sups | S Cells | S2 Cells | | | |
| Hu-1 | 1.00±0.00 | 0.60±0.06 | 0.40±0.06 | 0.59±0.05 | 0.41±0.05 | 1.01±0.01 | 1.00±0.00 | 1.00±0.00 |
| Delta | 1.06±0.05 | n.d. | n.d. | n.d. | n.d. | n.d. | 1.11±0.04 | 6.07±2.99 |
| BA.1 | 0.41±0.05 | 0.53±0.02 | 0.47±0.02 | 0.66±0.03 | 0.34±0.03 | 1.14±0.06 | 0.67±0.02 | 16.64±2.91 |
| BA.2 | 0.26±0.05 | 0.60±0.03 | 0.40±0.03 | 0.65±0.03 | 0.35±0.03 | 0.82±0.05 | 0.69±0.03 | 16.30±5.95 |
| G339D | 1.10±0.02 | 0.58±0.06 | 0.42±0.06 | 0.57±0.06 | 0.43±0.06 | n.d. | 1.16±0.09 | 2.77±1.06 |
| S373P | 0.81±0.14 | 0.60±0.08 | 0.40±0.08 | 0.67±0.07 | 0.33±0.07 | 0.97±0.04 | 1.06±0.11 | 4.43±1.50 |
| S375F | 0.10±0.00 | 0.92±0.04 | 0.08±0.04 | 0.87±0.07 | 0.13±0.07 | 0.54±0.07 | 0.15±0.00 | 1.45±0.77 |
| K417N | 1.06±0.02 | 0.54±0.07 | 0.46±0.07 | 0.64±0.06 | 0.36±0.06 | n.d. | 1.23±0.02 | 4.38±1.44 |
| N440K | 1.05±0.09 | 0.58±0.08 | 0.42±0.08 | 0.61±0.06 | 0.39±0.06 | n.d. | 1.30±0.08 | 3.94±1.29 |
| S477N | 0.99±0.06 | 0.60±0.08 | 0.40±0.08 | 0.65±0.05 | 0.35±0.05 | n.d. | 1.19±0.01 | 2.23±0.67 |
| T478K | 1.04±0.07 | 0.58±0.11 | 0.42±0.11 | 0.61±0.05 | 0.39±0.05 | n.d. | 1.21±0.01 | 2.18±1.39 |
| E484A | 0.82±0.10 | 0.51±0.15 | 0.49±0.15 | 0.61±0.05 | 0.39±0.05 | n.d. | 1.27±0.06 | 1.38±0.59 |
| Q493R | 1.02±0.03 | 0.60±0.05 | 0.40±0.05 | 0.59±0.06 | 0.41±0.06 | n.d. | 1.20±0.06 | 3.63±1.22 |
| Q498R | 1.03±0.08 | 0.64±0.05 | 0.36±0.05 | 0.63±0.08 | 0.37±0.08 | n.d. | 1.21±0.05 | 2.36±0.72 |
| N501Y | 0.98±0.07 | 0.68±0.06 | 0.32±0.06 | 0.61±0.08 | 0.39±0.08 | 1.22±0.07 | 1.10±0.10 | 3.39±1.19 |
| Y505H | 0.93±0.08 | 0.48±0.12 | 0.52±0.12 | 0.58±0.05 | 0.42±0.05 | n.d. | 1.20±0.05 | 2.94±1.02 |
| D614G | 1.20±0.07 | 0.65±0.07 | 0.35±0.07 | 0.52±0.05 | 0.48±0.05 | 0.81±0.08 | 1.36±0.04 | 2.58±0.79 |
| H655Y | 0.95±0.09 | 0.44±0.09 | 0.56±0.09 | 0.57±0.04 | 0.43±0.04 | n.d. | 1.18±0.05 | 1.47±0.36 |
| N679K | 0.73±0.15 | 0.57±0.07 | 0.43±0.07 | 0.56±0.07 | 0.44±0.07 | n.d. | 1.06±0.05 | 2.62±0.88 |
| P681H | 0.85±0.05 | 0.59±0.05 | 0.41±0.05 | 0.58±0.06 | 0.42±0.06 | n.d. | 1.18±0.02 | 2.37±0.65 |
| N764K | 1.12±0.06 | 0.34±0.07 | 0.66±0.07 | 0.55±0.04 | 0.45±0.04 | n.d. | 1.26±0.06 | 2.44±0.84 |
| D796Y | 0.82±0.08 | 0.68±0.04 | 0.32±0.04 | 0.63±0.09 | 0.37±0.09 | n.d. | 1.10±0.04 | 0.81±0.31 |
| Q954H | 0.70±0.01 | 0.73±0.07 | 0.27±0.07 | 0.70±0.08 | 0.30±0.08 | n.d. | 0.91±0.04 | 0.76±0.19 |
| N969K | 0.58±0.05 | 0.54±0.10 | 0.46±0.10 | 0.62±0.05 | 0.38±0.05 | n.d. | 0.79±0.02 | 1.23±0.31 |
| A67V | 0.75±0.03 | 0.56±0.05 | 0.44±0.05 | 0.61±0.04 | 0.39±0.04 | n.d. | 1.04±0.02 | 0.23±0.09 |
| Δ69-70 | 1.11±0.01 | 0.45±0.06 | 0.55±0.06 | 0.57±0.03 | 0.43±0.03 | n.d. | 1.29±0.04 | 0.52±0.13 |
| T95I | 1.13±0.03 | 0.44±0.07 | 0.56±0.07 | 0.52±0.05 | 0.48±0.05 | n.d. | 1.29±0.04 | 2.52±1.02 |
| Δ142-144 | 0.86±0.02 | 0.49±0.11 | 0.51±0.11 | 0.58±0.07 | 0.42±0.07 | n.d. | 1.05±0.04 | 4.22±1.87 |
| Y145D | 0.80±0.04 | 0.46±0.08 | 0.54±0.08 | 0.60±0.06 | 0.40±0.06 | n.d. | 1.09±0.05 | 6.98±2.45 |
| Δ211 | 0.76±0.03 | 0.55±0.12 | 0.45±0.12 | 0.64±0.06 | 0.36±0.06 | n.d. | 1.09±0.06 | 0.08±0.02 |
| 214EPE | 1.04±0.04 | 0.48±0.07 | 0.52±0.07 | 0.60±0.02 | 0.40±0.02 | n.d. | 1.18±0.02 | 6.95±2.09 |
| L212I | 1.13±0.02 | 0.65±0.06 | 0.35±0.06 | 0.56±0.04 | 0.44±0.04 | n.d. | 1.28±0.04 | 5.30±1.54 |
| S371L | 0.36±0.05 | 0.77±0.10 | 0.23±0.10 | 0.65±0.06 | 0.35±0.06 | 0.74±0.07 | 0.62±0.02 | 3.07±1.03 |
| G446S | 0.83±0.14 | 0.62±0.11 | 0.38±0.11 | 0.66±0.06 | 0.34±0.06 | n.d. | 1.19±0.05 | 1.25±0.39 |
| G496S | 1.00±0.05 | 0.57±0.08 | 0.43±0.08 | 0.58±0.06 | 0.42±0.06 | n.d. | 1.27±0.03 | 3.06±0.91 |
| T547K | 0.86±0.03 | 0.69±0.11 | 0.31±0.11 | 0.59±0.05 | 0.41±0.05 | n.d. | 1.14±0.04 | 1.42±0.35 |
| N856K | 0.44±0.09 | 0.54±0.09 | 0.46±0.09 | 0.57±0.03 | 0.43±0.03 | 0.82±0.13 | 0.99±0.11 | 1.18±0.40 |
| L981F | 1.20±0.08 | 0.60±0.17 | 0.40±0.17 | 0.68±0.03 | 0.32±0.03 | 0.80±0.17 | 1.50±0.04 | 0.96±0.41 |
| T19I | 0.53±0.07 | 0.59±0.25 | 0.41±0.25 | 0.76±0.06 | 0.24±0.06 | n.d. | 0.62±0.03 | 1.25±0.43 |
| Δ24-26 | 0.66±0.04 | 0.61±0.20 | 0.39±0.20 | 0.76±0.05 | 0.24±0.05 | n.d. | 0.64±0.02 | 2.25±1.68 |
| A27S | 1.00±0.07 | 0.63±0.03 | 0.37±0.03 | 0.62±0.02 | 0.38±0.02 | n.d. | 1.24±0.07 | 5.63±2.12 |
| G142D | 0.99±0.02 | 0.41±0.12 | 0.59±0.12 | 0.58±0.03 | 0.42±0.03 | n.d. | 1.22±0.03 | 4.54±2.58 |
| V213G | 1.06±0.05 | 0.52±0.09 | 0.48±0.09 | 0.58±0.03 | 0.42±0.03 | n.d. | 1.18±0.07 | 4.35±1.72 |
| S371F | 0.50±0.01 | 0.75±0.08 | 0.25±0.08 | 0.61±0.02 | 0.39±0.02 | 0.91±0.10 | 0.15±0.00 | 3.93±1.77 |
| T376A | 0.44±0.05 | 0.76±0.11 | 0.24±0.11 | 0.80±0.06 | 0.20±0.06 | 0.58±0.11 | 0.20±0.01 | 2.09±0.69 |
| D405N | 1.08±0.07 | 0.66±0.08 | 0.34±0.08 | 0.65±0.03 | 0.35±0.03 | n.d. | 1.26±0.06 | 1.58±0.97 |
| R408S | 1.12±0.06 | 0.60±0.05 | 0.40±0.05 | 0.62±0.03 | 0.38±0.03 | n.d. | 1.28±0.09 | 5.30±1.37 |
| SSS/LPF | 0.25±0.01 | n.d. | n.d. | n.d. | n.d. | 0.59±0.03 | 0.18±0.00 | n.d. |
| SSS/FPF | 0.28±0.03 | n.d. | n.d. | n.d. | n.d. | 0.76±0.17 | 0.20±0.00 | n.d. |

For each mutation in Spike analyzed, the table lists: normalized PP infection of CaCo-2 cells (Infection); levels of full-length Spike in supernatants (S Sups) and cells (S Cells); levels of S2 Spike subunit in supernatants (S2 Sups) and cells (S2 Cells); binding of Spike to ACE2 (ACE2 interaction); automated quantification of syncytia formation in HEK293T cells expressing the indicated mutant S proteins and Human ACE2 (Syncytia formation); average TCID50 values obtained for neutralization of the indicated mutant S proteins by sera from five vaccinated individuals relative to those obtained for the Hu-1 S (Serum neutralization). See methods for detail.

**Table S2 (related to Figure 6).** Origin and neutralizing activity of sera from BNT/BNT vaccinated individuals.

| Donor | AGE | Sex |
|---|---|---|
| 1 | 61 | w |
| 2 | 28 | w |
| 3 | 58 | m |
| 4 | 27 | w |
| 5 | 37 | m |

**WT**

| Donor | S Hu-1 | Delta | BA.1 | BA.2 |
|---|---|---|---|---|
| 1 | 0,15 | 0,69 | 3,88 | 0,7 |
| 2 | 0,9 | 1,52 | >10 | >10 |
| 3 | 0,4 | 7,11 | >10 | >10 |
| 4 | 0,99 | 3,36 | >10 | >10 |
| 5 | 0,29 | 0,82 | 5,44 | 6,03 |

**Colors:**

| |
|---|
| NTD |
| RBD |
| RBM |
| S1 |
| S2 |
| S2' |

**IC50 Values BA.1/BA.2**

| Donor | G339D | S373P | S375F | K417N | N440K | S477N | T478K | E484A | Q493R | Q498R |
|---|---|---|---|---|---|---|---|---|---|---|
| 1 | 0,29 | 0,88 | 0,17 | 1,23 | 1,09 | 0,61 | 0,46 | 0,51 | 0,89 | 0,6 |
| 2 | 1,07 | 0,97 | 0,29 | 0,87 | 0,73 | 0,55 | 0,12 | 0,2 | 0,34 | 0,55 |
| 3 | 1,1 | 1,82 | 0,31 | 2,79 | 2,15 | 1,28 | 2,9 | 0,78 | 1,86 | 1,54 |
| 4 | 6,73 | 9,11 | 4,43 | 4,4 | 5,33 | 2,49 | 0,1 | 1,14 | 6,07 | 2,57 |
| 5 | 0,34 | 0,42 | 0,16 | 0,4 | 0,27 | 0,22 | 0,1 | 0,07 | 0,31 | 0,22 |

| Donor | N501Y | Y505H | D614G | H655Y | N679K | P681H | N764K | D796Y | Q954H | N969K |
|---|---|---|---|---|---|---|---|---|---|---|
| 1 | 0,84 | 0,85 | 0,69 | 0,29 | 0,77 | 0,68 | 0,77 | 0,28 | 0,17 | 0,32 |
| 2 | 0,41 | 0,43 | 0,58 | 0,43 | 0,41 | 0,62 | 0,41 | 0,19 | 0,15 | 0,33 |
| 3 | 2,53 | 1,77 | 1,45 | 0,82 | 1,17 | 0,9 | 1,42 | 0,45 | 0,42 | 0,57 |
| 4 | 3,63 | 3,35 | 3,23 | 2,16 | 3,8 | 2,88 | 2,02 | 0,63 | 0,95 | 1,45 |
| 5 | 0,27 | 0,21 | 0,22 | 0,2 | 0,24 | 0,42 | 0,31 | 0,07 | 0,13 | 0,21 |

**IC50 Values BA.1**

| Donor | A67V | d69-70 | T95I | d142-144 | Y145D | d211 | 214EPE | L212I | S371L | G446S | G496S | T547K | N856K | L981F |
|---|---|---|---|---|---|---|---|---|---|---|---|---|---|---|
| 1 | 0,01 | 0,12 | 0,31 | 0,49 | 1,32 | 0,01 | 1,47 | 1,03 | 0,84 | 0,16 | 0,81 | 0,33 | 0,32 | 0,38 |
| 2 | 0,26 | 0,27 | 0,79 | 1,09 | 1,01 | 0,06 | 2,22 | 1,04 | 0,64 | 0,28 | 0,74 | 0,39 | 0,21 | 0,24 |
| 3 | 0,04 | 0,19 | 2,59 | 4,61 | 5,18 | 0,02 | 2,92 | 3,48 | 1,84 | 1,03 | 1,7 | 0,85 | 0,59 | 0,37 |
| 4 | 0,55 | 0,84 | 1,41 | 2,64 | 9,56 | 0,03 | 9,89 | 7,56 | 3,91 | 1,55 | 3,77 | 1,49 | 1,83 | 0,72 |
| 5 | 0,04 | 0,05 | 0,5 | 0,68 | 0,38 | 0,05 | 0,64 | 0,6 | 0,15 | 0,21 | 0,29 | 0,23 | 0,07 | 0,1 |

**IC50 Values BA.2**

| Donor | T19I | d24-26 | A27S | G142D | V213G | S371F | T376A | D405N | R408S |
|---|---|---|---|---|---|---|---|---|---|
| 1 | 0,16 | 0 | 1,94 | 0,43 | 0,31 | 0,36 | 0,39 | 0,04 | 1,25 |
| 2 | 0,22 | 1,08 | 0,96 | 0,92 | 0,93 | 0,21 | 0,5 | 0,44 | 1,96 |
| 3 | 0,75 | 0,11 | 1,92 | 5,88 | 2,74 | 2,66 | 1,17 | 0,06 | 2,96 |
| 4 | 2,51 | 8,79 | 7,39 | 2,18 | 9,74 | 9,24 | 3,88 | 1,64 | 6,73 |
| 5 | 0,15 | 0,25 | 0,56 | 0,53 | 0,55 | 0,27 | 0,12 | 1,53 | 0,52 |

**monoclonal ABs IC50**

| | WT | 21k | 21l | K417N | N440K | G446S | S477N | T478K | E484A | Q493R | G496S | Q498R | N501Y | Y505H |
|---|---|---|---|---|---|---|---|---|---|---|---|---|---|---|
| amlanivimab | 0,02667 | >20 | >20 | n.a | 0,02307 | 0,02157 | n.a | n.a | >20 | >20 | 0,02022 | n.a | n.a | n.a |
| Casivirimab | 0,03493 | >20 | >20 | 0,433 | n.a | n.a | 0,1565 | 0,1187 | 0,1395 | 0,08354 | 0,01754 | 0,09144 | n.a | 0,03691 |
| Imdevimab | 0,0985 | >20 | 13,7 | n.a | >20 | >20 | n.a. | n.a. | n.a. | n.a. | n.a. | 0,1605 | 0,1655 | n.a. |
