# Supplementary material for: Determinants of Spike infectivity, processing, and neutralization in SARS-CoV-2 Omicron subvariants BA.1 and BA.2
Source: Cell Host Microbe. 2022 Sep 14;30(9):1255–1268.e5. doi: 10.1016/j.chom.2022.07.006 (PMC9289044; doi:10.1016/j.chom.2022.07.006)
Supplement: Document S2. Article plus supplemental information [file mmc3.pdf]

Article

Cell Host & Microbe

Determinants of Spike infectivity, processing, and neutralization in SARS-CoV-2 Omicron subvariants BA.1 and BA.2

Graphical abstract

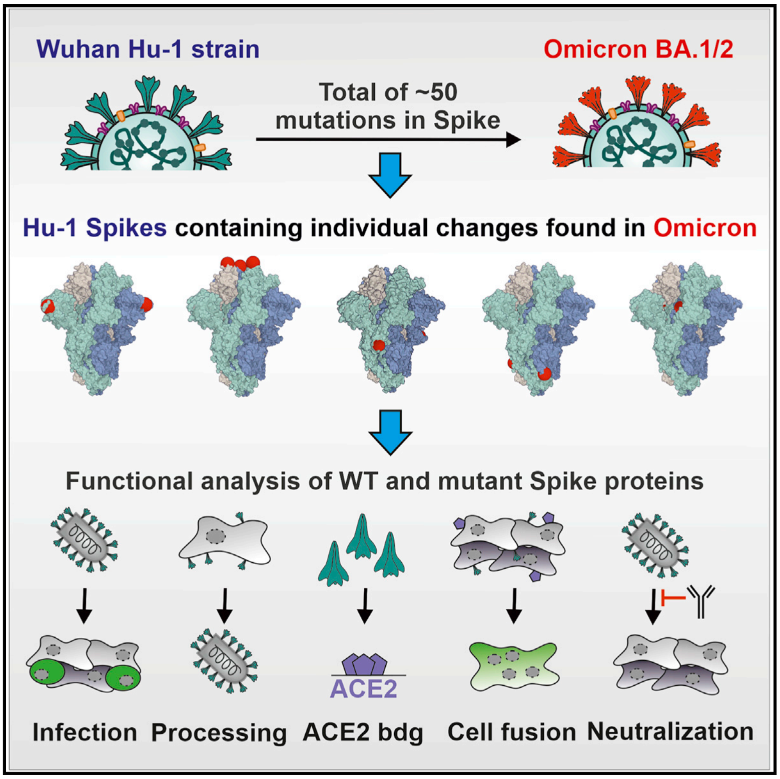

Highlights

- Omicron Spike residue changes of S371F/L, S373P, and S375F impair Spike function
- Changes of Q954H and N969K in HR1 reduce, while that of L981F enhances, S-mediated infection
- Omicron-specific mutations in the NTD and RBD of Spike reduce neutralization
- N440K, G446S, E484A, and Q493R confer resistance to bamlanivimab or imdevimab

Authors

Chiara Pastorio, Fabian Zech, Sabrina Noettger, ..., Theo Sanderson, Konstantin M.J. Sparrer, Frank Kirchhoff

Correspondence

frank.kirchhoff@uni-ulm.de

In brief

The Omicron Spike protein contains numerous mutations thought to play key roles in the efficient spread and immune evasion of this currently dominating SARS-CoV-2 variant. Pastorio, Zech, and colleagues examined the impact of mutations characteristic of the BA.1 and/or BA.2 Omicron lineages on Spike function, processing, and susceptibility to neutralization.

Pastorio et al., 2022, Cell Host & Microbe *30*, 1255–1268
September 14, 2022 © 2022 The Authors. Published by Elsevier Inc.
https://doi.org/10.1016/j.chom.2022.07.006

CellPress

Cell Host & Microbe

CellPress
OPEN ACCESS

Article

Determinants of Spike infectivity, processing, and neutralization in SARS-CoV-2 Omicron subvariants BA.1 and BA.2

Chiara Pastorio,[1,6] Fabian Zech,[1,6] Sabrina Noettger,[1] Christoph Jung,[2,3,4] Timo Jacob,[2] Theo Sanderson,[5] Konstantin M.J. Sparrer,[1] and Frank Kirchhoff[1,7,*]
[1]Institute of Molecular Virology, Ulm University Medical Centre, 89081 Ulm, Germany
[2]Institute of Electrochemistry, Ulm University, 89081 Ulm, Germany
[3]Electrochemical Energy Storage, Helmholtz-Institute-Ulm (HIU), 89081 Ulm, Germany
[4]Karlsruhe Institute of Technology (KIT), 76344 Karlsruhe, Germany
[5]Francis Crick Institute, London MW1 1AT, UK
[6]These authors contributed equally
[7]Lead contact
*Correspondence: frank.kirchhoff@uni-ulm.de
https://doi.org/10.1016/j.chom.2022.07.006

SUMMARY

SARS-CoV-2 Omicron rapidly outcompeted other variants and currently dominates the COVID-19 pandemic. Its enhanced transmission and immune evasion are thought to be driven by numerous mutations in the Omicron Spike protein. Here, we systematically introduced BA.1 and/or BA.2 Omicron Spike mutations into the ancestral Spike protein and examined the impacts on Spike function, processing, and susceptibility to neutralization. Individual mutations of S371F/L, S375F, and T376A in the ACE2-receptor-binding domain as well as Q954H and N969K in the hinge region 1 impaired infectivity, while changes to G339D, D614G, N764K, and L981F moderately enhanced it. Most mutations in the N-terminal region and receptor-binding domain reduced the sensitivity of the Spike protein to neutralization by sera from individuals vaccinated with the BNT162b2 vaccine and by therapeutic antibodies. Our results represent a systematic functional analysis of Omicron Spike adaptations that have allowed this SARS-CoV-2 variant to dominate the current pandemic.

INTRODUCTION

SARS-CoV-2, the causative agent of the coronavirus disease 2019 (COVID-19) pandemic, has infected more than 542 million people around the globe and caused about 6.3 million fatalities (https://coronavirus.jhu.edu/map.html; June 24, 2022). Effective vaccination is the best way to get this devastating pandemic under control. A variety of safe and effective vaccines against SARS-CoV-2 are available and more than 11.6 billion vaccine doses have been administered to date. However, low access to or acceptance of vaccines, together with the emergence of new SARS-CoV-2 variants, jeopardize this strategy. So-called variants of concern (VOCs) pose a particular risk. Their increased transmissibility, efficient immune evasion, and altered pathogenicity are mainly determined by the viral spike (S) protein (Harvey et al., 2021; Jung et al., 2022; Tao et al., 2021).

Currently, the fifth SARS-CoV-2 VOC, termed Omicron, dominates the COVID-19 pandemic. The Omicron VOC was initially detected in Botswana and South Africa in November 2021 and outcompeted the Delta VOC in an amazingly short time. Evolutionary studies revealed that the Omicron VOC evolved independently, possibly in a chronically infected immunocompromised individual, human population under poor surveillance, or an unknown non-human species from which it spilled back to humans (Karim et al., 2021; Wei et al., 2021). Omicron contains a strikingly high number of mutations (Jung et al., 2022), especially in its S protein, compared with other variants and the initial Wuhan strains. Recent studies support the finding that this VOC is highly transmissible and shows an increased ability to infect convalescent and vaccinated individuals (Altarawneh et al., 2022; Espenhain et al., 2021; Grabowski et al., 2022; Pulliam et al., 2021). This agrees with the finding that the Omicron VOC shows reduced susceptibility to neutralizing antibodies induced by previous SARS-CoV-2 infection or vaccination (Andrews et al., 2021; Cele et al., 2022; Hoffmann et al., 2021; Lu et al., 2021; Planas et al., 2021; Wilhelm et al., 2021). Notably, accumulating evidence suggests that Omicron infections are associated with milder symptoms and decreased hospitalization and fatality rates compared with infections with the Delta SARS-CoV-2 VOC (Moore and Baden, 2022; Wolter et al., 2022).

The SARS-CoV-2 S protein is the major membrane glycoprotein required for recognition of the viral receptor

This is an open access article under the CC BY license (http://creativecommons.org/licenses/by/4.0/).

angiotensin-converting enzyme 2 (ACE2) and subsequent entry into target cells (Hoffmann et al., 2020; Letko et al., 2020). Thus, the S protein critically determines the cell tropism and transmissibility of SARS-CoV-2 in human populations. To mediate attachment and fusion, the S precursor needs to be proteolytically processed by cellular proteases after synthesis. The proprotein convertase furin cleaves S at the S1/S2 site to generate the S1 subunit, which is responsible for receptor binding, while the transmembrane serine protease 2 (TMPRSS2), or cathepsins B and L, cleave at the S2′ site just upstream of the hydrophobic fusion peptide (FP) to release the S2 subunit mediating membrane fusion (Walls et al., 2020; Wrapp et al., 2020). In its active form, the S protein of SARS-CoV-2 forms trimers on the surface of viral particles. Consequently, the S protein is also the major target of protective humoral immune responses (Walls et al., 2020), and all currently licensed COVID-19 vaccines are based on the SARS-CoV-2 S antigen. Thus, mutations in the N-terminal domain or receptor-binding domain (NTD and RBD, respectively) of S can increase resistance to neutralizing antibodies (Cao et al., 2022; Dai and Gao, 2021; Dejnirattisai et al., 2022; McCallum et al., 2022; VanBlargan et al., 2021). It is clear that alterations in the S protein of the Omicron VOC play a key role in its high transmissibility, efficient immune evasion, and reduced pathogenicity. However, the impact of most amino acid changes distinguishing the Omicron S protein from that of the original Wuhan SARS-CoV-2 strain on viral infectivity and susceptibility to neutralization remains to be determined.

Here, we analyzed the functional impact of individual amino acid changes that distinguish the initial 21K (BA.1) Omicron VOC and the currently dominating 21L (BA.2) variant from the early 2020 Wuhan SARS-CoV-2 isolate. To achieve this, we introduced a total of 48 mutations in the S protein of the Wuhan strain and determined their impact on viral infectivity, expression, and proteolytic processing, as well as susceptibility to neutralizing antibodies and sera from vaccinated individuals. We show that several amino acid changes found in Omicron S proteins impair infectivity and demonstrate that numerous alterations in the NTD and RBD of BA.1 and/or BA.2 S proteins affect neutralization by sera from (BioNTech/BioNTech) BNT/BNT-vaccinated individuals and therapeutic antibodies.

RESULTS

Generation of S proteins containing mutations found in Omicron

Omicron is currently classified into two major lineages, BA.1 (21K) and BA.2 (21L) (Figure 1A; Hadfield et al., 2018; Sagulenko et al., 2018). BA.1 has replaced the Delta VOC and dominated the COVID-19 pandemic at the beginning of 2022 (Figure 1B). Subsequently, the BA.2 lineage has outcompeted BA.1 and is currently responsible for the majority of infections (Figure 1B). New variants are continuously emerging, and some of them (i.e., BA.2.12.1, BA.4, and BA.5) contain additional mutations in residues L452 and F486 of their Spike protein that may further increase immune evasion and transmission fitness (Cao et al., 2022). Although only ~13% of the SARS-CoV-2 genome encodes for the S protein, this region contains most mutations distinguishing the Omicron VOCs from the original Wuhan Hu-1 SARS-CoV-2 strain. Many of the mutations that differ in the Omicron S proteins from those of other SARS-CoV-2 variants are located in the RBD that interacts with the viral ACE2 receptor and is a major target of neutralizing antibodies (Figure 1C). The Omicron BA.1 and BA.2 S proteins share about 20 amino acid changes in S compared with the 2020 Wuhan Hu-1 strain, and 12 of these are located in the RBD (Figures 1C and 1D). In the consensus, a total of 14 mutations are specific for BA.1 and 9 for BA.2 (Figures 1C and 1D). Thirteen of the 23-S consensus lineage-specific variations are located in the NTD (Figure 1D). All 43 non-synonymous defining mutations, insertions, and deletions found in BA.1 and BA.2 Omicron VOCs (https://covariants.org/variants/21L) were introduced individually in the S protein of the original Wuhan Hu-1 strain by site-directed mutagenesis. Sequence analysis of the full-length S genes verified that all constructs contained the desired mutations (Figure 1D) and confirmed the absence of additional changes.

Impact of mutations in Omicron Spike on viral pseudoparticle infection

To analyze the functional impact of mutations found in the Omicron BA.1 and BA.2 variants, we generated vesicular stomatitis virus (VSV) particles pseudotyped with the parental and mutant SARS-CoV-2 S proteins. Previous studies established that these VSV pseudoparticles (VSVpps) mimic key features of SARS-CoV-2 entry, such as receptor usage, cell tropism, protease dependency, and susceptibility to neutralizing antibodies (Hoffmann et al., 2020; Riepler et al., 2020; Schmidt et al., 2016). We found that the BA.1 and BA.2 S showed significantly reduced infection efficiencies compared with the Wuhan Hu-1 S, while the S protein of the Delta VOC displayed significantly increased activity (Figure 2A, left). Notably, we used Spike proteins lacking an artificial deletion of the C-terminal ER-retention motif that is often used to increase SARS-CoV-2 pseudoparticle infectivity for *in vitro* assays (Yu et al., 2021). Instead, we used full-length S proteins containing a V5 epitope tag for unbiased analysis of expression and processing. Most of the 20 amino acid changes in S that are shared between the BA.1 and BA.2 variants did not significantly affect the efficiency of VSVpp infection (Figures 2A and S1). In agreement with previous findings (Korber et al., 2020; Yurkovetskiy et al., 2020), substitution of D614G slightly enhanced infection. Similarly, mutations of G339D and N764K had subtle enhancing effects. Notably, modest enhancing effects were not due to a saturation of infection because only up to 10% of all target cells became GFP positive during the single round of infection (Figure S1). Substitution of S375F in the RBD drastically impaired, and mutations of Q954H and N969K in heptad repeat 1 (HR1) reduced, VSVpp infectivity (Figures 2A and S1).

Most of the BA.1 and BA.2 specific variations in the NTD of the S protein had minor effects on VSVpp infectivity (Figure 2A). Three changes (Δ69-70, T95I, and L212I) in the NTD slightly enhanced, and six alterations (T19I, Δ24-26, A67V, Δ142-144, Y145D, and Δ211) reduced, VSVpp infection. Similar to the shared S375F, mutations of S371L or S371F in the BA.1 and BA.2 S proteins, respectively, strongly impaired viral infectivity. The adjacent BA.2-specific T376A change had similar disruptive effects (Figure 2A). Mutation of N856K that is specific for BA.1 and might stabilize the FP proximal region (Zhang et al., 2022a, 2022b), and T19I or Δ24-26 near the N terminus of BA.2 S,

CellPress
OPEN ACCESS

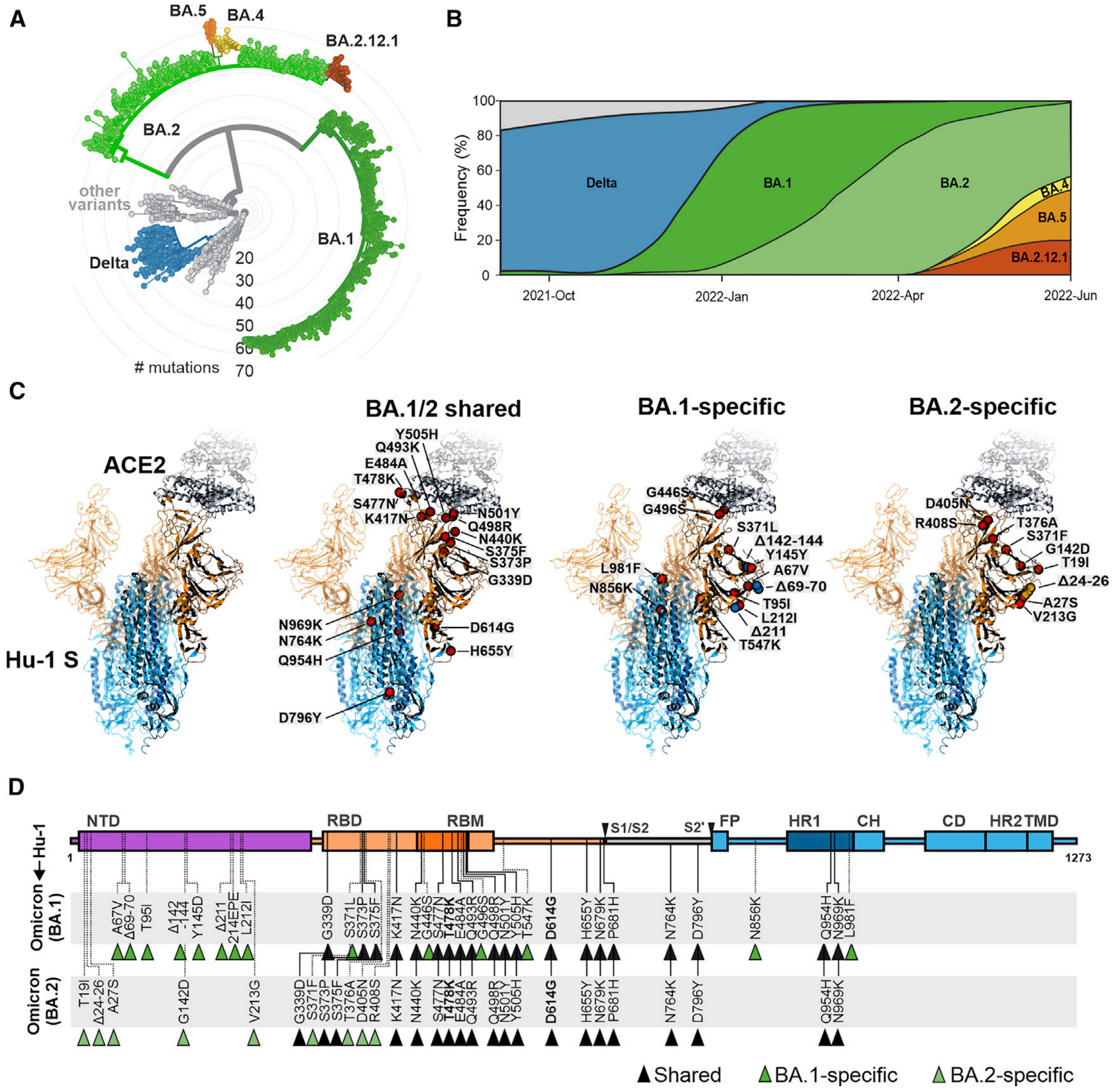

Figure 1. Features of Omicron BA.1 and BA.2 VOCs

(A) Radial phylogenetic tree of representative SARS-CoV-2 strains (n = 2,793 genomes, sampled between December 2019 and June 2022), scaled according to their divergence, compared with the Wuhan Hu-1 sequence. Retrieved from Nextstrain on June 24, 2022 (https://nextstrain.org/ncov/open/global/6m?l=radial) and modified. Color coding according to VOCs as indicated.

(B) Frequencies of SARS-CoV-2 Delta, BA.1, and BA.2 sequences in data from GenBank from September 2021 to June 2022. Scaled to 100%. Retrieved and modified from Nextstrain on June 24, 2022. Blue, Delta VOC; green, BA.1; light green, BA.2; yellow, BA.4; orange, BA.5; and red, BA.2.12.1.

(C) Overview of the SARS-CoV-2 spike structure (downloaded from PDB: 7KNB) and localization of amino acid changes that are shared between BA.1 and BA.2 or specific for BA.1 or BA.2 as indicated. S1 (orange), S2 (blue), ACE2 (gray), mutations (red), BA.1-specific deletions (blue), BA.2-specific deletions (yellow).

(D) Schematic depiction of the SARS-CoV-2 spike, its domains, and amino acid alterations in Omicron BA.1 (green) and BA.2 (light green) VOCs compared with the Wuhan Hu-1 sequence. S1 subunit: N-terminal domain, NTD (purple); receptor-binding domain, RBD (orange). Receptor-binding motif, RBM (dark orange). S2 subunit: fusion peptide, FP (blue); heptad repeat 1, HR1 (dark blue); central helix, CH; connector domain, CD; heptad repeat 2, HR2; transmembrane domain, TM (blue).

markedly reduced VSVpp infection (Figure 2A), although these residues do not affect known functional domains.

To assess infection kinetics and to challenge the above-mentioned infection results, we performed assays allowing automated quantification of the number of VSVpp-infected (GFP+) Caco-2 cells over time. The various mutant S proteins mediated infection with similar kinetics but varying and frequently reduced efficiencies (Figure 2B). The results confirmed that the BA.1 and BA.2 S show diminished infection efficiency compared with the Hu-1 Wuhan S protein. Individual mutations of T19I, Δ24-26, S371L, S375F, T376A, and N856K all reduced the activity of the Hu-1 S to levels similar or below that obtained for the BA.1 S protein (Figure 2B). In contrast, shared mutations of N440K and D614G, as well as BA.1-specific changes of Δ69-70, Δ211, insertion of 214EPE, and mutation of L981F, increased infection efficiencies. Our results agree with recent findings

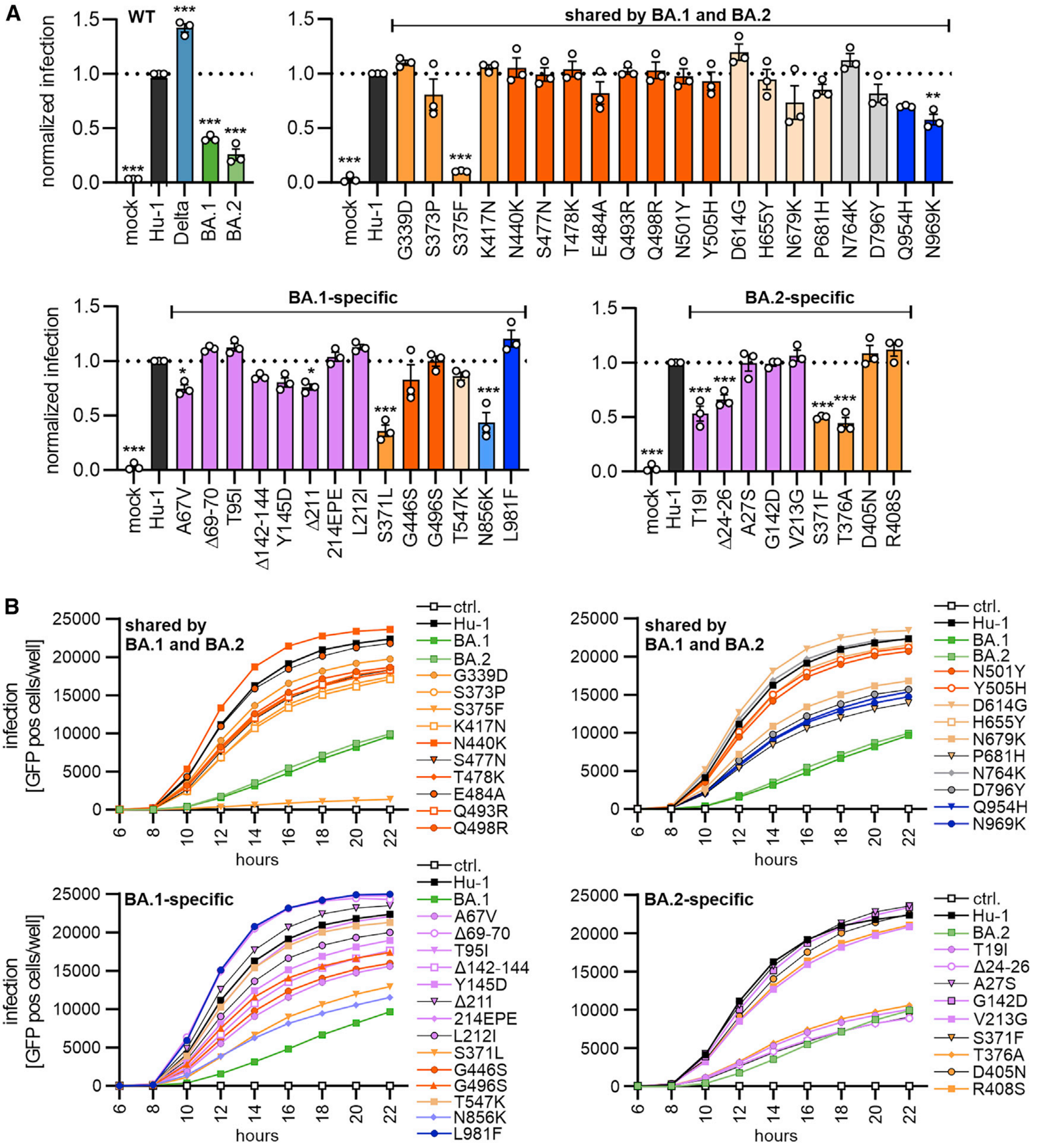

Figure 2. Impact of mutations in Omicron on Spike-mediated infection

(A) Automatic quantification of infection events of CaCo-2 cells transduced with VSVΔG-GFP pseudotyped with SARS-CoV-2 Hu-1 (gray), Delta (blue), BA.1 (green), BA.2 (light green), or indicated mutant S proteins. The localization of each mutation in S is indicated by color. S1: NTD (purple), RBD (orange), RBM (dark orange), and others (light orange). S2: HR1 (dark blue) and others (light blue). Bars represent the mean of three independent experiments (± SEM). Statistical significance was tested by one-way ANOVA. *p < 0.05; **p < 0.01; ***p < 0.001.

(B) Infection kinetics of CaCo-2 cells infected by VSVpp containing the indicated mutant S proteins. Infected GFP+ cells were automatically quantified over a period of 22 h.

See also Figure S1.

suggesting that the Q954H and N969K changes in HR1 reduce rather than enhance fusion efficiency (Suzuki et al., 2022; Xia et al., 2022; Zhao et al., 2022). In addition, our analysis revealed that N856K in BA.1 S and T19I, as well as Δ24-26 in the BA.2 NTD, strongly impaired S-mediated infection. Perhaps most notably, all individual mutations in the three serine residues in a small loop region (S371L/F, S373P, S375F), as well as the adjacent BA.2-specific T376A change, severely impaired S-mediated infection.

Inefficient processing and virion incorporation of specific Spike variants

To examine expression, proteolytic processing, and virion incorporation of the mutant S proteins, we performed

CellPress
OPEN ACCESS

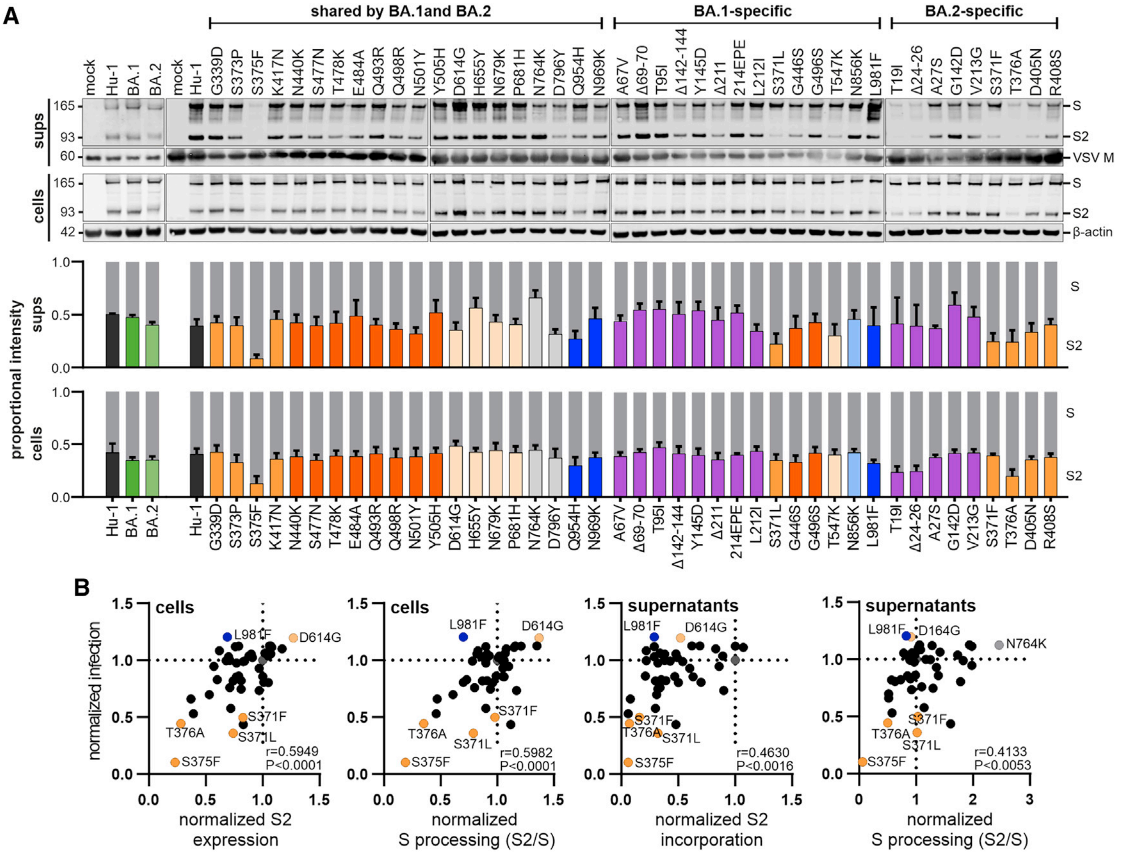

Figure 3. Expression and processing of Spike proteins containing mutations present in the Omicron BA.1 and BA.2 VOCs

(A) The upper panels show exemplary immunoblots of whole cells lysates (WCLs) and VSVpp containing supernatants of HEK293T cells transfected with vectors expressing the Hu-1, BA.1, BA.2, or mutant SARS-CoV-2 S proteins and infected with VSVΔG-GFP. Blots were stained with anti-V5 (Spike), anti-ß-actin, and anti-VSV-M protein. Lower panels: expression levels of uncleaved, full-length Spike protein (S, gray bars) and the S2 subunit (bars colored according to the corresponding domains, as shown in Figure 1D) were quantified. The results show mean values (±SEM) obtained from three independent experiments.

(B) Correlation of the S2 expression/incorporation and S2/S processing of the parental S Hu-1 or indicated mutant S proteins in cells and supernatants, with the corresponding pseudotype infection rates. The correlation coefficients (r values) and two-tailed p values are provided.

See also Figure S2.

comprehensive western blot analyses of extracts of HEK293T cells infected with VSVΔG-eGFP and transfected with S expression constructs and the S-containing VSVpp in the culture supernatants. In agreement with the infectivity data, most individual amino acid changes, deletions, or insertions had no significant impact on S expression and processing (Figure 3A). All 46 parental and mutant full-length S proteins were readily detected in the cellular extracts (Figure 3A). However, the mutations in S371L, S373P, S375F, and T376A that impaired S infectivity (Figure 2) also reduced the efficiency of processing and/or incorporation into viral pseudoparticles (Figure 3A). The phenotypes of the S375F and T376A mutants were most striking, and these S variants were hardly processed. Two BA.2 specific alterations in S (T19I and Δ24-26) that were less active in infection assays were associated with reduced levels of S protein on VSVpp (Figure 3A). Altogether, the levels of S2 protein expression and processing in cellular extracts relative to the parental Hu-1 S proteins correlated well with one another (Figure S2A) and with the efficiency of S-mediated infection (Figure 3B, left). Similar but less significant correlations were observed for VSVpp infection and the Spike levels in the culture supernatants (Figure 3B, right). T19I, Δ24-26, S375F, and T376A reduced the levels of both S & S2 incorporated into VSVpp, while S371L/F mainly affected S2 levels in the particles. In comparison, mutations of Q493R, T547K, D796Y, and N856K reduced VSVpp infection without exerting significant effects on S expression and processing in the cells, although T547K and D796Y were associated with reduced levels of S2 in VSVpp (Figure 3). None of the mutations (H655Y, N679K, and P681H) located near the S1/S2 cleavage site had significant effects on S processing (Figure 3). In addition, confocal microscopy showed that mutant S proteins showing enhanced (D614G, L981F) or impaired (T19I, S371L/F, S373P, S375F, T376A) activity, all localized at the cell surface (Figure S2B), indicating that disruptive effects were not due to impaired trafficking or mislocalization. Altogether, our results revealed that changes of T19I, Δ24-26, T376A, S375F, and Q954H reduce VSVpp infectivity by affecting S processing, although they are not located in proximity to the S1/S2 furin cleavage site.

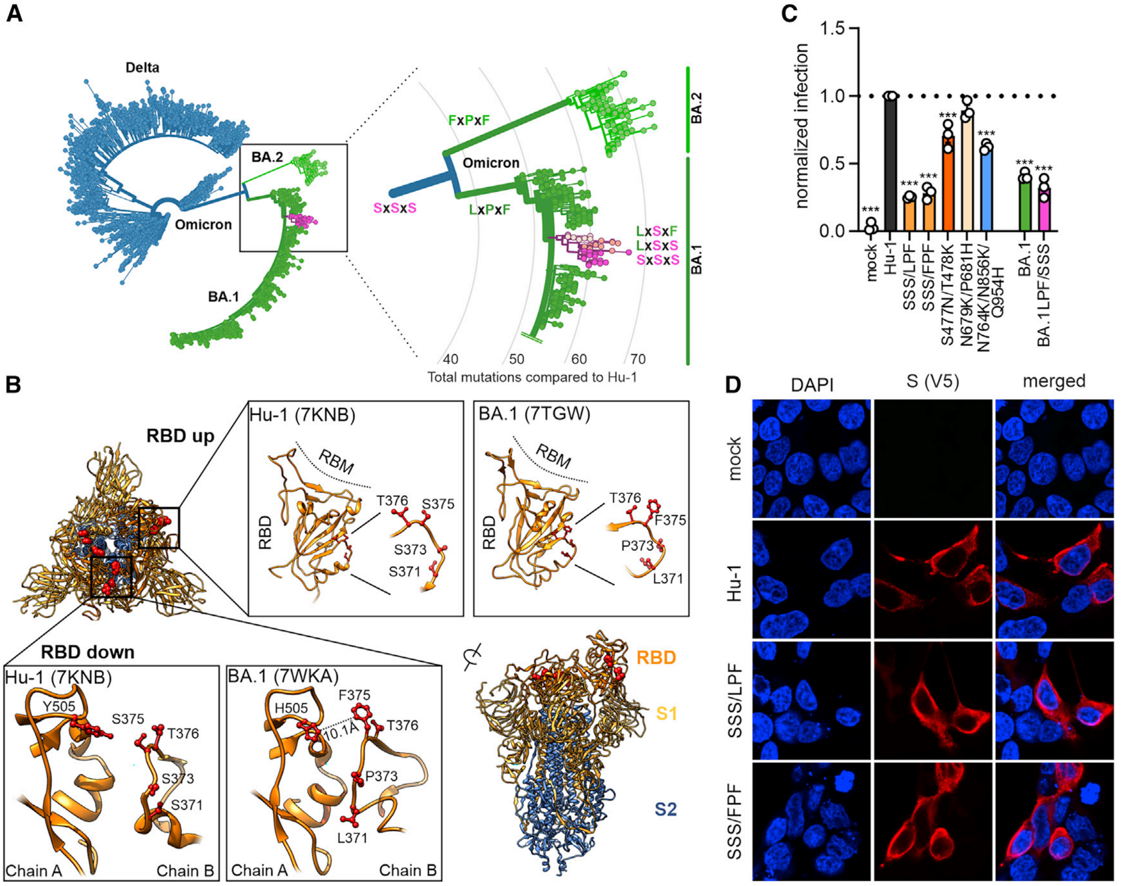

Figure 4. Functional relevance of S371L/F, S373P, and S375F changes in the Spike protein

(A) Phylogenetic tree of Delta and Omicron BA.1 and BA.2 strains. Amino acids at position 371, 373, and 375 are indicated; all other SARS-CoV-2 variants almost invariantly contain three serines at these positions. Color coding as indicated according to VOC. Retrieved and modified from Nextstrain on April 7, 2022.

(B) Close-up view of the region encompassing the mutations S371L, S373P, and S375F and the surrounding region. Downloaded from PDB: 7KNB, 7TGW, or 7WKA as indicated.

(C) Automatic quantification of infection events of CaCo-2 cells transduced with VSVΔG-GFP pseudotyped with SARS-CoV-2 Hu-1 or indicated combined mutations. Bars represent the mean of three independent experiments (±SEM). Statistical significance was tested by one-way ANOVA. *p < 0.05; **p < 0.01; ***p < 0.001.

(D) Immunofluorescence images of HEK293T cells expressing the parental S Hu-1, the BA.1-specific SSSxLPF, or the BA.2-specific SSSxFPF mutations. Scale bars, 10 μm.

See also Figure S3.

Functional relevance of serine mutations in an RBD loop region

It came as a surprise that all individual mutations of S371, S373, and S375 that are found in the Wuhan Hu-1 strains and the Alpha, Beta, Gamma, and Delta VOCs to 371L/F, 373P, and 375F present in Omicron, severely impaired S function. Analysis of available SARS-CoV-2 sequences revealed that the BA.1 and BA.2 S proteins usually contain combined changes of SxSxS to LxPxF or FxPxF, respectively (Figures 4A and S3A). However, we identified a small subcluster within the BA.1 sequences showing apparent reversions to serine residues (Figure 4A). Altogether, about 0.55% of all 3.7 million available Omicron Spike sequences (∼20,000 in total) report a serine at amino acid position 371, 373, and/or 375 (Figure S3A). Closer examination of the underlying sequencing data revealed, however, that most if not all of these sequences show very poor coverage in the corresponding region (representative examples shown in Figure S3B). These profound losses of coverage suggest that these sequencing runs were unable to properly identify the residue at this position. Thus, serine residues at position 371, 373, and/or 375 of Omicron Spike proteins appear to be due to faulty next generation sequencing (NGS) sequence processing rather than genuine reversions to residues found in the S proteins of other SARS-CoV-2 variants.

The serine-containing loop is located adjacent to the RBD and might affect its up and down state (Sztain et al., 2021) as well as RBD-RBD interactions (Wrobel et al., 2022; Figure 4B). In agreement with this, recent structural analyses showed that the changes of SxSxS to LxPxF or FxPxF favor the inactive down conformation of the RBD, particularly in the BA.2 Spike protein (Gobeil et al., 2022; Stalls et al., 2022). Altogether, these results raised the possibility that the combination of S371-S373-S375 or

CellPress
OPEN ACCESS

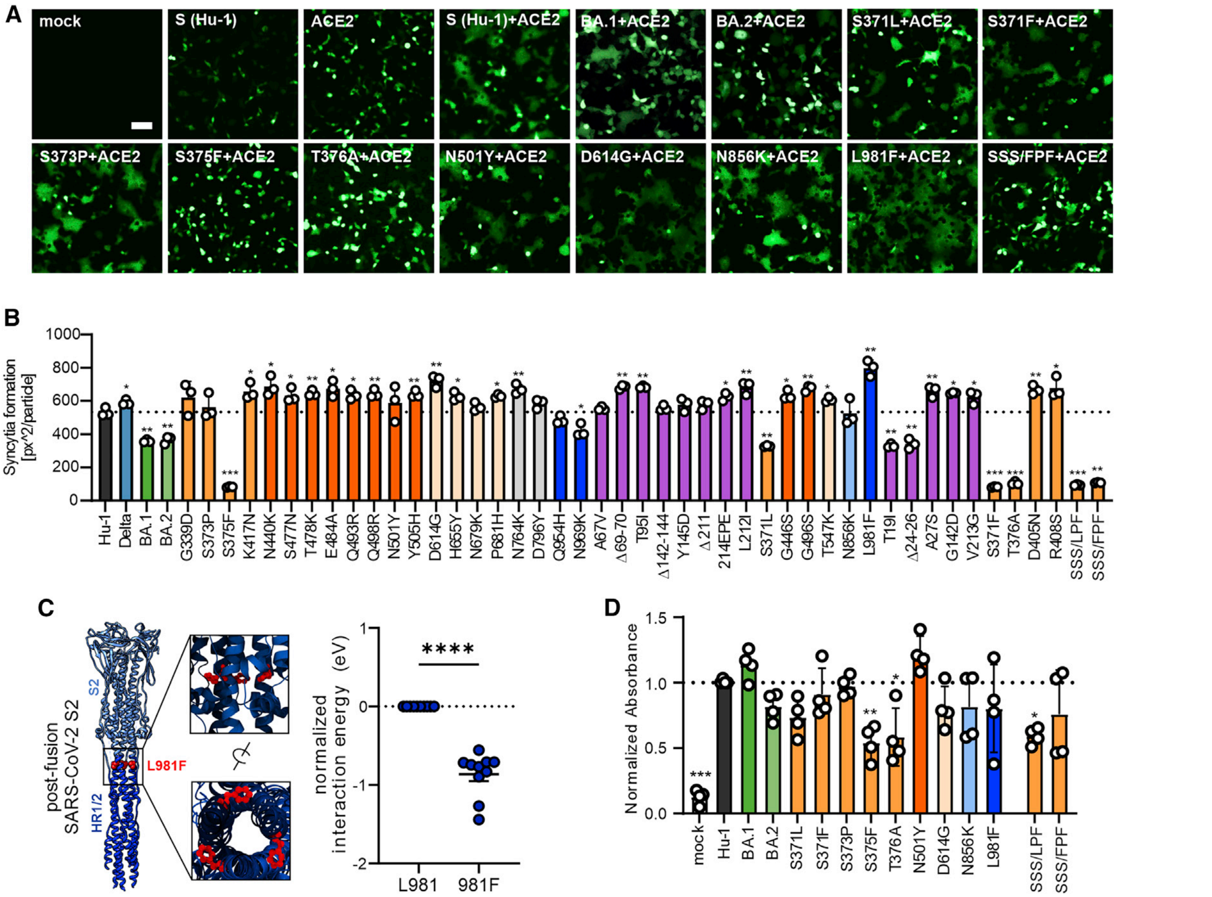

Figure 5. Impact of mutations in Omicron Spike on cell-to-cell fusion and ACE2 interaction

(A) Representative fluorescence microscopy images of HEK293T cells expressing parental Hu-1 or indicated mutant S proteins, human ACE2, and GFP (green). Scale bar, 125 μm.

(B) Automatic quantification of syncytia formation of HEK293T cells expressing parental Hu-1 or indicated mutant S proteins and human ACE2. Bars represent the mean of three independent experiments (±SEM). Statistical significance was tested by two-tailed Student's t test with Welch's correction. *$p < 0.05$; **$p < 0.01$; ***$p < 0.001$.

(C) Overview on the SARS-CoV-2 post-fusion spike structure (downloaded from PDB: 6M3W) and comparative ReaxFF simulation of the mutation L981F.

(D) Binding of the indicated Hu-1 and mutant S proteins to ACE2 binding using whole-cell lysates of transfected HEK293T. Bars represent the mean of three independent experiments (±SEM). Statistical significance was tested by one-way ANOVA. *$p < 0.05$; **$p < 0.001$.

See also Table S1.

371F/L373P375F might be required for effective S function and processing. To address this experimentally, we generated the LPF (BA.1) and FPF (BA.2) triple mutants of the Hu-1 S protein, as well as a BA.1 mutant S containing changes of LPF to SSS, and analyzed their ability to mediate VSVpp infection. All showed substantially lower fusion activity than the Hu-1 S and the presence of the three serine residues did not enhance the fusion activity of the BA.1 S (Figure 4C). In comparison, combined changes of S477N/T478K in the RBD and N764K/N856K/Q954H in S2 had only modest disruptive effects and alterations of N679K/P681H near the S2′ processing site did not significantly change the infection efficiency of the Hu-1 S protein (Figure 4C). Intracellular localization analyses showed that the LxPxF and FxPxF mutant S proteins were readily detectable at the cell surface, just like the parental Hu-1 S protein (Figure 4D). Thus, in agreement with the results on individual mutations (Figure S2B), the impaired activity and processing of the triple mutant S proteins is not due to altered trafficking or subcellular localization but might involve an inactive conformation.

Effect of mutations in Omicron Spike on cell-to-cell fusion and ACE2 interaction

To determine the fusogenic activity of our library of Spike proteins, we quantified cell-to-cell fusion of HEK293T cells expressing wild-type (WT) or mutant S proteins and ACE2. We found that the co-expression of human ACE2 and the parental Hu-1, as well as most mutant S proteins, resulted in efficient formation of large syncytia (Figures 5A and 5B). In contrast, the S371F, S375F, T376A, and triple LxPxF or FxPxF mutant S proteins did not lead to detectable fusion, while intermediate phenotypes were observed for the T19I, Δ24-26 and S371L, and Spikes. In agreement with the VSVpp infection data (Figure 2), the parental BA.1 and BA.2 S proteins were less active than the Hu-1 S, and individual or combined mutations in the SxSxS motif disrupted the

ability of the Hu-1 S protein to mediate membrane fusion (summarized in Table S1).

Mutations of D614G and (to a stronger extent) L981F significantly increased syncytia formation (Figures 5A and 5B). L981 is located in the HR1 region of the S2 protein that interacts with HR2 to form a six-helix bundle to drive virus-host or cell-to-cell membrane fusion (Figure 5C). In agreement with the functional data, molecular modeling of HR1/HR2 interactions using reactive force field simulations predicted that the mutation of L981F significantly enhances interactions between HR1 and HR2 (Figure 5C). Taken together, syncytia formation is promoted by the D614G found in all VOCs and the Omicron-specific mutation L981F, but almost abrogated by S371F, S375F, T376A, and the triple SSS to LPF or FPF changes.

To examine the impact of specific mutations in the Omicron S protein on ACE2 interaction, we used a previously established *in vitro* S-ACE2 binding assay (Zech et al., 2021). Immobilized ACE2 is incubated with lysates of transfected HEK293T cells transfected with mutant S expression constructs. The S protein retained after washing is detected by a mouse αV5-Ab and quantified using a secondary HRP-conjugated anti-mouse Ab. The S371F, S373P, D614G, N856K, and L981F mutations in the Hu-1 S had little if any effect on S binding to human ACE2 (Figure 5D). In comparison, individual substitutions of S375F and T376A and the triple mutations (SSS to LPF or FPF) reduced the levels of S protein bound to ACE2 (Figure 5A). In line with published data (Tian et al., 2021), the mutation of N501Y enhanced binding of the SARS-CoV-2 S protein to human ACE2 (Figure 5D).

Mutations in the Omicron S affect neutralization by sera from immunized individuals

Numerous recent studies have shown that the Omicron BA.1 and BA.2 Spikes show reduced sensitivity to neutralizing antibodies (Abs) induced upon infection and vaccination (Andrews et al., 2021; Cele et al., 2022; Hoffmann et al., 2021; Lu et al., 2021; Zhang et al., 2022a, 2022b). To determine the contribution of individual amino acid changes to immune evasion by Omicron, we compared the sensitivity of the four parental variants—Hu-1, Delta, BA.1, and BA.2—with 43 mutant S proteins, each harboring one Omicron-specific mutation, to neutralization by sera from five individuals who received a prime boost vaccination with the mRNA-based BioNTech-Pfizer (BNT162b2) vaccine (Table S2). This vaccine has been approved in 141 countries (https://covid19.trackvaccines.org/vaccines/6/), is frequently used in Europe and the US, and induces efficient protection against most COVID-19 variants (Polack et al., 2020) but shows about a 5- to 40-fold lower efficiency against Omicron (Cele et al., 2022; Collie et al., 2022; Iketani et al., 2022; Lu et al., 2021). Predictably, five randomly selected sera collected 2 weeks after the second dose of BNT neutralized BA.1 and BA.2 with substantially lower efficiency on average than the original Wuhan Hu-1 and Delta variants (Figure 6A, left; Table S2). A variety of shared as well as BA.1- or BA.2-specific amino acid changes reduced sensitivity to neutralization (examples shown in Figure 6A). The mutations, deletions, and insertions in the NTD of Omicron S are associated with significant structural changes (Zhang et al., 2022a, 2022b) and contribute to immune evasion of the Omicron VOC (Cao et al., 2022; Dejnirattisai et al., 2022; McCallum et al., 2022; VanBlargan et al., 2021). Our analyses revealed that most individual mutations found in the NTD of BA.1 and BA.2 S proteins reduced neutralization sensitivity (Figure 6B). Deletion of residues 142–144 in BA.1 and G142D in BA.2 had the strongest effects (~9-fold reduction) followed by mutation of Y145D and 214EPE (both in BA.1) that conferred ~7-fold resistance. Amino acid changes in the RBD, such as G339D, S371L, S373P, K417N, and N440K, as well as BA.2-specific alterations of S371F and R408S, reduced sensitivity to neutralization by BNT/BNT sera, usually in the range of ~2- to 5-fold (Figure 6B). In comparison, five of the six mutations in the S2 region had little if any effect on neutralization. Only the N764K change reduced it on average by about 2-fold. Altogether, 27 of the 43 mutations analyzed enhanced antibody-mediated neutralization resistance by >2-fold (Figure 6B). This further supports the finding that a large number of substitutions in the Omicron Spike cooperate to allow efficient viral evasion of humoral immune responses.

In the final set of experiments, we examined the impact of specific mutations in the Omicron S protein on neutralization sensitivity to the U.S. Food and Drug Administration (FDA)-approved therapeutic monoclonal antibodies REGN10987 (marketed as imdevimab), LY-CoV555 (marketed as bamlanivimab), and REGN10933 (marketed as casivirimab) that all target the receptor-binding-motif (RBM) domain (Figure 7A). The BA.1 VOC was not inhibited by imdevimab, and the N440K or G446S mutations in the Hu-1 S were sufficient to confer full resistance (Figure 7B). In comparison, BA.2 S showed some remaining susceptibility to imdevimab, and changes of Q498R and N501Y had little effect. Both BA.1 and BA.2 were fully resistant to bamlanivimab, and substitutions of E484A or Q493R were sufficient to confer resistance (Figure 7B). These results agree with those of two recent studies that also examined the impact of individual amino acid changes found in the BA.1 and BA.2 spikes on neutralization by a panel of monoclonal antibodies (Iketani et al., 2022; Liu et al., 2022). Finally, casivirimab showed little if any activity against BA.1 and BA.2 but neutralized all mutant S proteins analyzed, albeit with lower efficacy compared with the original Hu-1 S (Figure 7B). Altogether, our results show that a strikingly high number of amino acid changes in the NTD and RBD regions of the Omicron S proteins contribute to evasion from neutralizing antibodies. The impact of individual mutations on susceptibility to neutralization varies strongly between sera obtained from individuals who received the BNT/BNT vaccine.

DISCUSSION

The Omicron VOC has outcompeted the previously dominating Delta VOC in an amazingly short time. It is generally accepted that the high number of changes in the Omicron S confer increased transmission efficiency and escape from neutralizing antibodies and are the main reason for effective spread of this VOC. Here, we systematically analyzed the functional impact of all individual amino acid changes, deletions, and insertions that are characteristic of the Omicron BA.1 and BA.2 VOCs. In total, we examined 48 mutant Spike constructs containing amino acid changes distinguishing BA.1 and BA.2 Omicron VOCs from the original Hu-1 Wuhan strain. We identified several changes that strongly impair Spike-mediated infection and proteolytic

CellPress
OPEN ACCESS

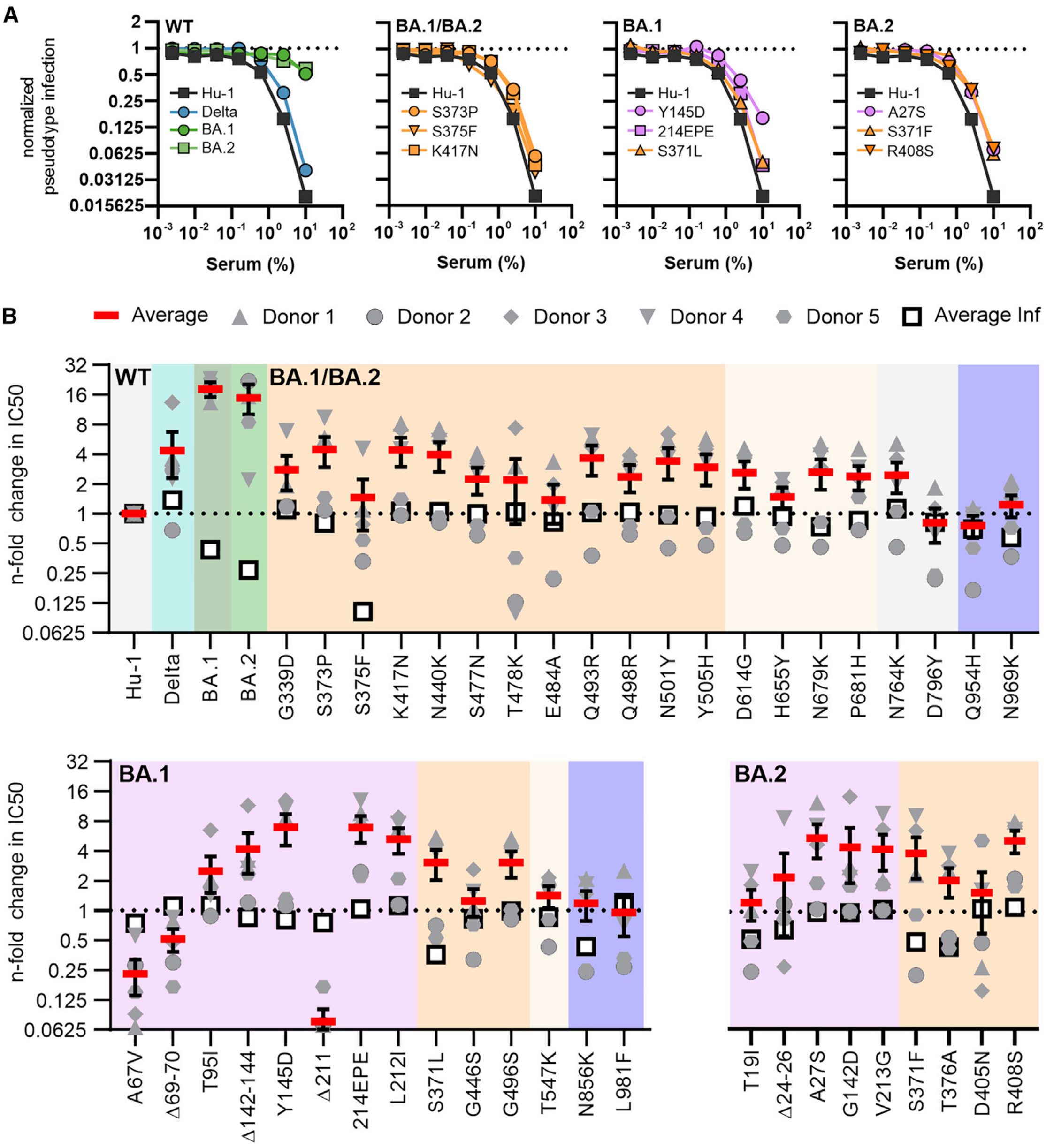

Figure 6. Impact of mutations in the Omicron Spike on serum neutralization

(A) Neutralization of VSVpp carrying the indicated wild-type and mutant S proteins by sera obtained from five BNT/BNT-vaccinated individuals compared with the untreated control (set to one). Shown are mean values obtained for the five sera, each tested in two technical replicates.

(B) Changes in $TCID_{50}$ values obtained for neutralization of the indicated mutant S proteins by sera from five vaccinated individuals relative to those obtained for the Hu-1 S. Solid red bars indicate mean values (±SEM) for the five sera and open black squares the average infectivity of the respective S-containing VSVpp shown in Figure 2A.

See also Table S2.

processing. In addition, we demonstrated that BA.1- or BA.2-specific mutations in the NTD, as well as shared alterations in the RBD, significantly reduce the susceptibility of Spike-containing VSVpp to neutralization by sera from BNT/BNT-vaccinated individuals and therapeutic antibodies.

One striking finding was that individual mutations of S371F/L, S375F, T376A, and (to a lesser extent) S373P in the RBD strongly impair Spike-containing pseudoparticle infectivity and Spike processing. S375F had the most drastic effect and almost fully disrupted Spike function and processing. This agrees with a recent preprint (Yamasoba et al., 2022) and is of particular interest because it has recently been reported that the S371L and S371F mutations in BA.1 and BA2, respectively, have major effects on neutralization by different RBD classes (Liu et al., 2022; Iketani et al., 2022). However, our results show that although these mutations significantly reduce S-mediated

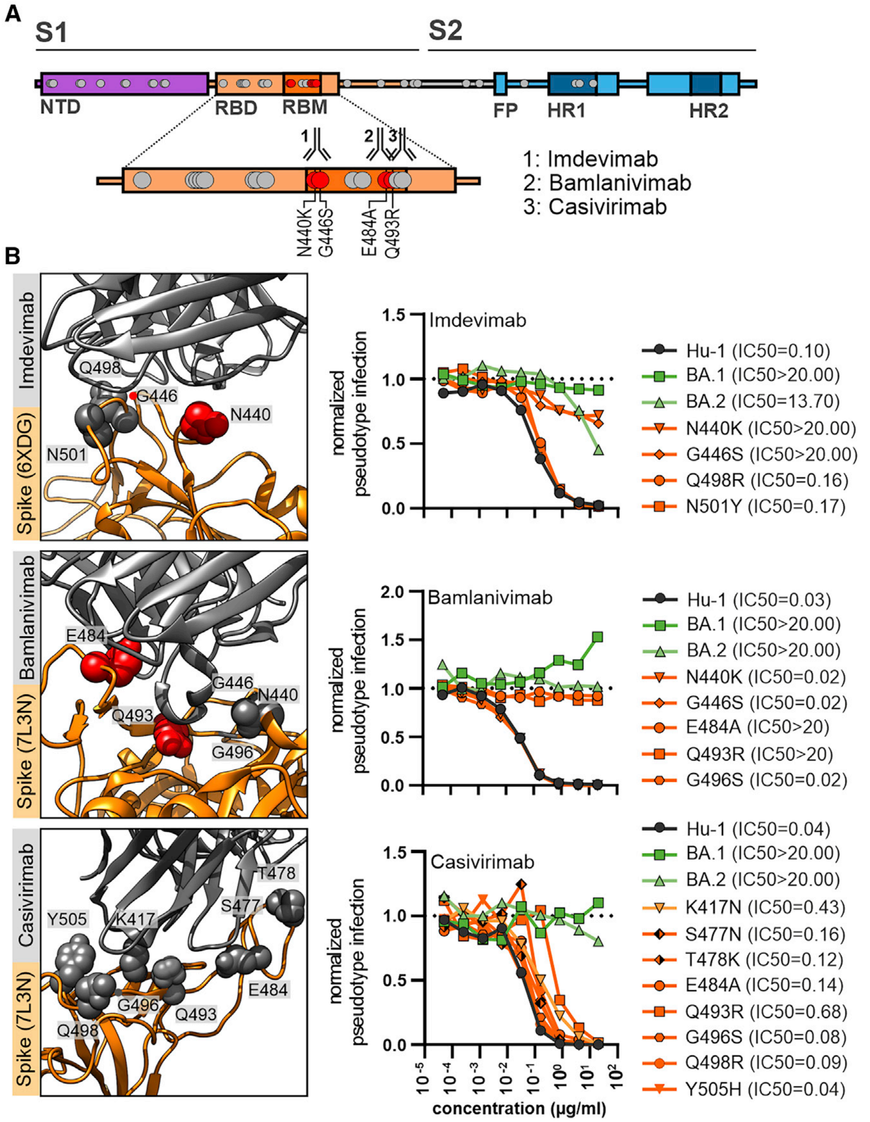

Figure 7. Impact of mutations in the Omicron Spike on neutralization by therapeutic Abs

(A) Schematic depiction of SARS-CoV-2 Spike domains, interaction sites of therapeutic antibodies, and resistance-conferring amino acid alterations highlighted in red.

(B) Close-up view of neutralizing antibodies binding the SARS-CoV-2 Spike (PDB: 6XDG or 7L3N as indicated) and automated quantification of GFP fluorescence of Caco-2 cells infected with VSVΔG-GFP pseudotyped with the indicated S variants. VSVpp were pre-treated (30 min, 37°C) with the indicated amounts of imdevimab, bamlanivimab or casivirimab. Lines represent the mean of three independent experiments. IC_{50} values indicate Ab concentrations (µg/mL) required to reduce pseudoparticle infection by 50%.

infection (Figure 2), they have only modest effects on neutralization by BNT/BNT sera (Figure 6). Notably, all these residues are part of a loop that may affect the open and closed conformation of the RBD. Recent structural analyses show that mutations of S371L, S373P, and S375F promote interprotomer interactions between the "down" RBDs. Specifically, it has been proposed that the S373P substitution induces conformational changes of the loop, resulting in closer packing of the RBD-RBD interface via interactions of S373P and S375F with the N501Y and Y505H substitutions in the adjacent RBD (Gobeil et al., 2022). Our functional analyses show that mutations of S371L/F and T376A severely affect Spike function, while changes of N501Y and Y505H have no disruptive effects on S-mediated infection. Both individual and combined mutations in the three serine residues (S371, S373, and S375) severely impaired the ability of the Hu-1 Spike protein to mediate virus-cell and cell-cell fusion. Although further studies are necessary, this agrees with the results of structural studies suggesting that changes of S371L, S373P, S375F, and perhaps T376A, might stabilize the inactive closed conformation of the Spike protein (Gobeil et al., 2022; Stalls et al., 2022).

The severe disruptive effects of mutations in the SxSxS region raised the possibility that these changes might revert in a subset of Omicron variants. Indeed, we found that about 0.5% of available Omicron S sequences contain apparent reversions of LxPxF to SxSxS (Figures 4A and S3A). Closer examination revealed, however, a profound loss of coverage in the region of interest in these sequences (Figure S4B), most likely resulting from a loss of sequencing amplicons due to mutations in the primer sites in the Spike coding region. This should lead to random nucleotides (Ns) being called at these positions, but the calling of a reference sequence can occur either due to misconfiguration or to low level contamination with, for example, a reference sequence from neighboring positive control wells. Alternatively, misconfigured processing pipelines can also call reference sequences inappropriately. Altogether, our analyses suggest that the vast majority of Omicron sequences in global databases that appear to encode S residues at position 371, 373, or 375 sites represent errors. This adds to the recent evidence that some apparent variations in the Spike proteins of newly emerging SARS-CoV-2 variants result from sequencing artifacts (Sanderson and Barrett, 2021) and that it is important to consider this possibility when assessing alterations in particular regions.

A variety of mutations in the S1 subunit (ΔH69/V70, T95I, ΔY144, K417N, T478K, N501Y, D614G, H655Y, and P681H) of BA.1 and/or BA.2 Omicron S proteins have previously been observed in other VOCs (Golcuk et al., 2021). As previously suggested (Mannar et al., 2021), we found that the deletion of ΔH69/V70 increased S-mediated infectivity (Figure 2). Our analyses also confirmed (Lam et al., 2021) that the mutation of N501Y in the RBM of the Omicron Spike increases the binding affinity to

ACE2 (Figure 5A). It has been proposed that mutations of H655Y, N679K, and P681H may increase furin-mediated S1/S2 cleavage and enhance pseudoparticle infectivity (Aggarwal et al., 2021; Cameroni et al., 2021; VanBlargan et al., 2021). We found that none of these three changes significantly enhanced S-mediated VSVpp infection (Figure 2) and only H655Y clearly enhanced S processing (Figure 3). Mutations of K417N, Q493R, Q498R, and N501Y are identical or similar to changes emerging during SARS-CoV-2 adaptation to experimentally infected mice (Huang et al., 2021; Sun et al., 2021) and were proposed to stabilize the RBD and ACE2 interaction (Meng et al., 2022) or to contribute to the ability of Omicron to infect mouse cells (Hoffmann et al., 2021). However, individual changes had no significant effect on VSVpp infectivity or processing but reduced susceptibility to BNT/BNT neutralization. Similarly, based on cryo-EM analyses, it has been suggested that alterations of Q493R, G496S, and Q498R in the RBD of the Omicron S form may allow the formation of stronger interactions with ACE2 that compensate for the disruptive effect of K417N (Mannar et al., 2021), but none of these four mutations individually significantly affected S-mediated infection (Figure 2).

Predictably, many shared mutations in the RBD domain of BA.1 and BA.2 S proteins reduced the sensitivity of VSVpp to neutralization by sera from BNT/BNT-vaccinated individuals (Figure 6A). In addition, we also found that mutations of N440K or G446S conferred resistance to imdevimab and changes of E484A or Q493R to bamlanivimab, respectively (Figure 7). This was expected as these mutations are located within the epitopes bound by these antibodies. Our results add to the evidence (Iketani et al., 2022; Liu et al., 2022) that single amino acid changes may confer full resistance to neutralizing antibodies. In comparison, the mutation of E484A that has also been observed in other SARS-CoV-2 VOCs and was suggested to be associated with immune escape (Rath et al., 2022) had only marginal effects on neutralization sensitivity. Unexpectedly, most lineage-specific changes in the NTD, such as A27S, T95I, Δ142-144, G142D, INS214EPE, L212I, and V213G, were at least as effective in reducing S sensitivity to neutralization by sera from BNT/BNT-vaccinated individuals as changes in the RBD (Figure 6A). This adds to the accumulating evidence that the NTD is an important target for neutralizing antibodies in sera from vaccinated individuals.

Three mutations (Q954H, N969K, and L981F) are located in the HR1 region of the S2 subunit of the S protein (Figure 1D). It was initially proposed that these changes might promote 6-helix bundle formation and subsequent fusion (Sarkar et al., 2021), but more recent evidence suggests that they may attenuate rather than enhance S-mediated fusion efficiency (Suzuki et al., 2022). In agreement with the latter, changes of Q954H and N969K clearly reduced S-mediated VSVpp infection (Figure 2). In contrast, the substitution of L981F enhanced Spike-mediated VSVpp infection and (more strongly) cell-to-cell fusion (Figures 5A and 5B). Reactive force simulations suggest that the L981F mutation enhances interactions between the HR1 and HR2 regions that drive fusion. Notably, recent data showed that the three mutations in the HR1 region of the Omicron S do not alter the global architecture of the post-fusion six-helix bundle (Yang et al., 2022), and peptide-based pan-CoV fusion inhibitors derived from the HR region maintain high potency against the SARS-CoV-2 Omicron VOC (Xia et al., 2022).

The molecular mechanisms of several mutations in the Omicron S protein remain to be fully elucidated. For example, BA.2-specific changes of T19I and Δ24-26 in the NTD severely reduced S-mediated infection and processing, although they do not affect known functional domains. It has been suggested that a shared mutation of N764K and a BA.2-specific substitution of N856K generate potential cleavage sites for SKI-1/S1P protease and might impede the exposition of the FP for membrane fusion (Maaroufi, 2022). We found that N764K is indeed associated with increased infectivity and increased levels of processed Spike in VSVpp. In comparison, N856K clearly reduced S-mediated infection despite normal processing.

Limitations of the study

In the present study, we used pseudotyped viral particles instead of replication-competent recombinant SARS-CoV-2 variants, which serves as a proxy to assess infectivity, fusion activity, and incorporation. In addition, the impact of many changes might be context-dependent, and this might explain why some individual changes had disruptive effects on Hu-1 S function although they are found in Omicron S proteins. It is difficult to predict which of the numerous mutations in the Omicron S might compensate for disruptive mutations. In addition, we analyzed only a limited number of sera from individuals who received a single vaccine regimen (BNT/BNT) and just a few therapeutic antibodies. Although further studies are required to fully understand the full consequences of all the complex changes in the Omicron Spike on viral infectivity, tropism, transmission, and pathogenesis, our results provide important insights into the functional impact of mutations characteristic for the Omicron VOC Spike that currently dominates the pandemic.

STAR★METHODS

Detailed methods are provided in the online version of this paper and include the following:

- KEY RESOURCES TABLE
- RESOURCE AVAILABILITY
  - Lead contact
  - Materials availability
  - Data and code availability
- EXPERIMENTAL MODEL AND SUBJECT DETAILS
  - Cell culture
  - Sera from vaccinated individuals
- METHOD DETAILS
  - Expression constructs
  - Molecular dynamics simulation
  - Pseudoparticle production
  - Infection assay
  - Pseudoparticle inhibition
  - Whole-cell and cell-free lysates
  - SDS-PAGE and immunoblotting
  - ACE2 interaction assay
  - Immunofluorescence
  - Quantification of syncytia formation
  - NGS sequence analysis
- QUANTIFICATION AND STATISTICAL ANALYSIS

SUPPLEMENTAL INFORMATION

Supplemental information can be found online at https://doi.org/10.1016/j.chom.2022.07.006.

ACKNOWLEDGMENTS

We thank Kerstin Regensburger, Regina Burger, Jana-Romana Fischer, Birgit Ott, Martha Meyer, Nicola Schrott, and Daniela Krnavek for technical assistance and Dorota Kmiec for critical reading of our manuscript. The ACE2 expression vector and original SARS-CoV-2 BA.1, BA.2, and B_1_617_2 Spike expression plasmids were kindly provided by Shinji Makino, Stefan Pöhlmann, and Beatrice H. Hahn, respectively. C.P. and S.N. are part of the International Graduate School for Molecular Medicine (IGradU). This study was supported by DFG grants to F.K. (CRC 1279 and SPP 1923), K.M.J.S. (CRC 1279, SPP 1923, and SP 1600/6-1), and T.J. (CRC 1279). F.K. and K.M.J.S. were supported by the BMBF (Restrict SARS-CoV-2 and IMMUNOMOD). T.S. was supported by the Wellcome Trust (210918/Z/18/Z).

AUTHOR CONTRIBUTIONS

C.P. and F.Z. performed most experiments with support from S.N. F.Z., K.M.J.S., and F.K. conceived the study and planned experiments. C.J. and T.J. performed molecular modeling and dynamics simulations. T.S. performed bioinformatic analysis of apparent reversions. F.K. wrote the initial draft of the manuscript. All authors reviewed and approved the manuscript.

DECLARATION OF INTERESTS

The authors declare no competing interests.

Received: April 25, 2022
Revised: June 29, 2022
Accepted: July 12, 2022
Published: July 18, 2022

REFERENCES

Aggarwal, A., Stella, A.O., Walker, G., Akerman, A., Milogiannakis, V., Brilot, F., Amatayakul-Chantler, S., Roth, N., Coppola, G., Schofield, P., et al. (2021). SARS-CoV-2 Omicron: evasion of potent humoral responses and resistance to clinical immunotherapeutics relative to viral variants of concern. Preprint at medRxiv. https://doi.org/10.1101/2021.12.14.21267772.

Altarawneh, H.N., Chemaitelly, H., Hasan, M.R., Ayoub, H.H., Qassim, S., AlMukdad, S., Coyle, P., Yassine, H.M., Al-Khatib, H.A., Benslimane, F.M., et al. (2022). Protection against the omicron variant from previous SARS-CoV-2 infection. N. Engl. J. Med. *386*, 1288–1290. https://doi.org/10.1056/NEJMc2200133.

Andrews, N., Stowe, J., Kirsebom, F., Toffa, S., Rickeard, T., Gallagher, E., Gower, C., Kall, M., Groves, N., O'Connell, A.-M., et al. (2021). Effectiveness of COVID-19 vaccines against the Omicron (B.1.1.529) variant of concern. N. Engl. J. Med. *386*, 1532–1546. https://www.nejm.org/doi/10.1056/NEJMoa2119451.

Bernstein, F.C., Koetzle, T.F., Williams, G.J., Meyer, E.F., Brice, M.D., Rodgers, J.R., Kennard, O., Shimanouchi, T., and Tasumi, M. (1977). The Protein Data Bank: a computer-based archival file for macromolecular structures. J. Mol. Biol. *112*, 535–542. https://doi.org/10.1016/s0022-2836(77)80200-3.

Cameroni, E., Saliba, C., Bowen, J.E., Rosen, L.E., Culap, K., Pinto, D., Marco, A.D., Zepeda, S.K., Iulio, J., di, Zatta, F., et al. (2021). Broadly neutralizing antibodies overcome SARS-CoV-2 Omicron antigenic shift. Nature *602*, 664–670.

Cao, Y., Yisimayi, A., Jian, F., Song, W., Xiao, T., Wang, L., Du, S., Wang, J., Li, Q., Chen, X., et al. (2022). BA.2.12.1, BA.4 and BA.5 escape antibodies elicited by Omicron infection. Nature. https://doi.org/10.1038/s41586-022-04980-y.

Cele, S., Jackson, L., Khoury, D.S., Khan, K., Moyo-Gwete, T., Tegally, H., San, J.E., Cromer, D., Scheepers, C., Amoako, D.G., et al. (2022). Omicron extensively but incompletely escapes Pfizer BNT162b2 neutralization. Nature *602*, 654–656.

Chen, C., Nadeau, S., Yared, M., Voinov, P., Xie, N., Roemer, C., and Stadler, T. (2021). CoV-Spectrum: analysis of globally shared SARS-CoV-2 data to identify and characterize new variants. Bioinformatics *38*, 1735–1737. https://doi.org/10.1093/bioinformatics/btab856.

Collie, S., Champion, J., Moultrie, H., Bekker, L.-G., and Gray, G. (2022). Effectiveness of BNT162b2 vaccine against omicron variant in South Africa. N. Engl. J. Med. *386*, 494–496. https://doi.org/10.1056/NEJMc2119270.

Dai, L., and Gao, G.F. (2021). Viral targets for vaccines against COVID-19. Nat. Rev. Immunol. *21*, 73–82. https://doi.org/10.1038/s41577-020-00480-0.

Dejnirattisai, W., Huo, J., Zhou, D., Zahradník, J., Supasa, P., Liu, C., Duyvesteyn, H.M.E., Ginn, H.M., Mentzer, A.J., Tuekprakhon, A., et al. (2022). SARS-CoV-2 Omicron-B.1.1.529 leads to widespread escape from neutralizing antibody responses. Cell *185*, 467–484.e15. https://doi.org/10.1016/j.cell.2021.12.046.

Espenhain, L., Funk, T., Overvad, M., Edslev, S.M., Fonager, J., Ingham, A.C., Rasmussen, M., Madsen, S.L., Espersen, C.H., Sieber, R.N., et al. (2021). Epidemiological characterisation of the first 785 SARS-CoV-2 Omicron variant cases in Denmark, December 2021. Euro Surveill. *26*, 2101146. https://doi.org/10.2807/1560-7917.ES.2021.26.50.2101146.

Gobeil, S.M.-C., Henderson, R., Stalls, V., Janowska, K., Huang, X., May, A., Speakman, M., Beaudoin, E., Manne, K., Li, D., et al. (2022). Structural diversity of the SARS-CoV-2 Omicron spike. Mol. Cell *82*, 2050–2068.e6. https://doi.org/10.1016/j.molcel.2022.03.028.

Golcuk, M., Yildiz, A., and Gur, M. (2021). The omicron variant increases the interactions of SARS-CoV-2 spike glycoprotein with ACE2. Preprint at bioRxiv. https://doi.org/10.1101/2021.12.06.471377.

Grabowski, F., Kochańczyk, M., and Lipniacki, T. (2022). The spread of SARS-CoV-2 variant Omicron with the doubling time of 2.0–3.3 days can be explained by immune evasion. Viruses *14*, 294. https://doi.org/10.3390/v14020294.

Hadfield, J., Megill, C., Bell, S.M., Huddleston, J., Potter, B., Callender, C., Sagulenko, P., Bedford, T., and Neher, R.A. (2018). Nextstrain: real-time tracking of pathogen evolution. Bioinformatics *34*, 4121–4123. https://doi.org/10.1093/bioinformatics/bty407.

Harvey, W.T., Carabelli, A.M., Jackson, B., Gupta, R.K., Thomson, E.C., Harrison, E.M., Ludden, C., Reeve, R., Rambaut, A., COVID-19 Genomics UK (COG-UK) Consortium, et al. (2021). SARS-CoV-2 variants, spike mutations and immune escape. Nat. Rev. Microbiol. *19*, 409–424. https://doi.org/10.1038/s41579-021-00573-0.

Hoffmann, M., Kleine-Weber, H., Schroeder, S., Krüger, N., Herrler, T., Erichsen, S., Schiergens, T.S., Herrler, G., Wu, N.-H., Nitsche, A., et al. (2020). SARS-CoV-2 cell entry depends on ACE2 and TMPRSS2 and is blocked by a clinically proven protease inhibitor. Cell *181*, 271–280.e8. https://doi.org/10.1016/j.cell.2020.02.052.

Hoffmann, M., Krüger, N., Schulz, S., Cossmann, A., Rocha, C., Kempf, A., Nehlmeier, I., Graichen, L., Moldenhauer, A.-S., Winkler, M.S., et al. (2021). The Omicron variant is highly resistant against antibody-mediated neutralization: implications for control of the COVID-19 pandemic. Cell *185*, 447–456.e11. https://doi.org/10.1016/j.cell.2021.12.032.

Huang, K., Zhang, Y., Hui, X., Zhao, Y., Gong, W., Wang, T., Zhang, S., Yang, Y., Deng, F., Zhang, Q., et al. (2021). Q493K and Q498H substitutions in Spike promote adaptation of SARS-CoV-2 in mice. EBiomedicine *67*, 103381. https://doi.org/10.1016/j.ebiom.2021.103381.

Humphrey, W., Dalke, A., and Schulten, K. (1996). VMD: visual molecular dynamics. J. Mol. Graphics *14*, 33–38.

Iketani, S., Liu, L., Guo, Y., Liu, L., Chan, J.F.-W., Huang, Y., Wang, M., Luo, Y., Yu, J., Chu, H., et al. (2022). Antibody evasion properties of SARS-CoV-2 Omicron sublineages. Nature *604*, 553–556. https://doi.org/10.1038/s41586-022-04594-4.

Jung, C., Kmiec, D., Koepke, L., Zech, F., Jacob, T., Sparrer, K.M.J., and Kirchhoff, F. (2022). Omicron: what makes the latest SARS-CoV-2 variant of

CellPress
OPEN ACCESS

concern so concerning? J. Virol. *96*, e0207721. https://doi.org/10.1128/jvi.02077-21.

Karim, F., Moosa, M.Y.S., Gosnell, B.I., Cele, S., Giandhari, J., Pillay, S., Tegally, H., Wilkinson, E., San, J.E., Msomi, N., et al. (2021). Persistent SARS-CoV-2 infection and intra-host evolution in association with advanced HIV infection. Preprint at medRxiv. https://doi.org/10.1101/2021.06.03.21258228.

Korber, B., Fischer, W.M., Gnanakaran, S., Yoon, H., Theiler, J., Abfalterer, W., Hengartner, N., Giorgi, E.E., Bhattacharya, T., Foley, B., et al. (2020). Tracking changes in SARS-CoV-2 spike: evidence that D614G increases infectivity of the COVID-19 virus. Cell *182*, 812–827.e19. https://doi.org/10.1016/j.cell.2020.06.043.

Lam, S.D., Waman, V.P., Orengo, C., and Lees, J. (2021). Insertions in the SARS-CoV-2 spike N-terminal domain may aid COVID-19 transmission. Preprint at bioRxiv. https://doi.org/10.1101/2021.12.06.471394.

Letko, M., Marzi, A., and Munster, V. (2020). Functional assessment of cell entry and receptor usage for SARS-CoV-2 and other lineage B betacoronaviruses. Nat. Microbiol. *5*, 562–569. https://doi.org/10.1038/s41564-020-0688-y.

Liu, L., Iketani, S., Guo, Y., Chan, J.F.-W., Wang, M., Liu, L., Luo, Y., Chu, H., Huang, Y., Nair, M.S., et al. (2022). Striking antibody evasion manifested by the Omicron variant of SARS-CoV-2. Nature *602*, 676–681. https://doi.org/10.1038/s41586-021-04388-0.

Lu, L., Mok, B.W.-Y., Chen, L.-L., Chan, J.M.-C., Tsang, O.T.-Y., Lam, B.H.-S., Chuang, V.W.-M., Chu, A.W.-H., Chan, W.-M., Ip, J.D., et al. (2021). Neutralization of SARS-CoV-2 Omicron variant by sera from BNT162b2 or Coronavac vaccine recipients. Clin. Infect. Dis. https://doi.org/10.1093/cid/ciab1041.

Maaroufi, H. (2022). The N764K and N856K mutations in SARS-CoV-2 Omicron BA.1 S protein generate potential cleavage sites for SKI-1/S1P protease. Preprint at bioRxiv. https://doi.org/10.1101/2022.01.21.477298.

Mannar, D., Saville, J.W., Zhu, X., Srivastava, S.S., Berezuk, A.M., Tuttle, K., Marquez, C., Sekirov, I., and Subramaniam, S. (2021). SARS-CoV-2 omicron variant: ACE2 binding, cryo-EM structure of spike protein-ACE2 complex and antibody evasion. Science *375*, 760–764.

McCallum, M., Czudnochowski, N., Rosen, L.E., Zepeda, S.K., Bowen, J.E., Walls, A.C., Hauser, K., Joshi, A., Stewart, C., Dillen, J.R., et al. (2022). Structural basis of SARS-CoV-2 Omicron immune evasion and receptor engagement. Science *375*, 864–868. https://doi.org/10.1126/science.abn8652.

Meng, B., Ferreira, I.A.T.M., Abdullahi, A., Goonawardane, N., Saito, A., Kimura, I., Yamasoba, D., Gerba, P.P., Fatihi, S., Rathore, S., et al. (2022). SARS-CoV-2 Omicron spike mediated immune escape and tropism shift. Nature *603*, 706–714.

Moore, P.L., and Baden, L.R. (2022). Omicron — decoupling infection from severe disease. N. Engl. J. Med. *386*, 1361–1362. https://doi.org/10.1056/NEJMe2201812.

Planas, D., Veyer, D., Baidaliuk, A., Staropoli, I., Guivel-Benhassine, F., Rajah, M.M., Planchais, C., Porrot, F., Robillard, N., Puech, J., et al. (2021). Reduced sensitivity of SARS-CoV-2 variant Delta to antibody neutralization. Nature *596*, 276–280. https://doi.org/10.1038/s41586-021-03777-9.

Polack, F.P., Thomas, S.J., Kitchin, N., Absalon, J., Gurtman, A., Lockhart, S., Perez, J.L., Pérez Marc, G., Moreira, E.D., Zerbini, C., et al. (2020). Safety and efficacy of the BNT162b2 mRNA Covid-19 vaccine. N. Engl. J. Med. *383*, 2603–2615. https://doi.org/10.1056/NEJMoa2034577.

Pulliam, J.R.C., Schalkwyk, C. van, Govender, N., Gottberg, A. von, Cohen, C., Groome, M.J., Dushoff, J., Mlisana, K., and Moultrie, H. (2021). Increased risk of SARS-CoV-2 reinfection associated with emergence of the Omicron variant in South Africa. Science *376*, eabn4947. https://doi.org/10.1126/science.abn4947.

Rath, S.L., Padhi, A.K., and Mandal, N. (2022). Scanning the RBD-ACE2 molecular interactions in Omicron variant. Biochem. Biophys. Res. Commun. *592*, 18–23. https://doi.org/10.1016/j.bbrc.2022.01.006.

Riepler, L., Rössler, A., Falch, A., Volland, A., Borena, W., von Laer, D., and Kimpel, J. (2020). Comparison of four SARS-CoV-2 neutralization assays. Vaccines (Basel) *9*, 13. https://doi.org/10.3390/vaccines9010013.

Sagulenko, P., Puller, V., and Neher, R.A. (2018). TreeTime: maximum-likelihood phylodynamic analysis. Virus Evol. *4*, vex042. https://doi.org/10.1093/ve/vex042.

Sanderson, T., and Barrett, J.C. (2021). Variation at Spike position 142 in SARS-CoV-2 Delta genomes is a technical artifact caused by dropout of a sequencing amplicon. Wellcome Open Res. *6*, 305. https://doi.org/10.12688/wellcomeopenres.17295.1.

Sarkar, R., Lo, M., Saha, R., Dutta, S., and Chawla-Sarkar, M. (2021). S glycoprotein diversity of the Omicron variant. Preprint at medRxiv. https://doi.org/10.1101/2021.12.04.21267284.

Schmidt, F.I., Lu, A., Chen, J.W., Ruan, J., Tang, C., Wu, H., and Ploegh, H.L. (2016). A single domain antibody fragment that recognizes the adaptor ASC defines the role of ASC domains in inflammasome assembly. J. Exp. Med. *213*, 771–790. https://doi.org/10.1084/jem.20151790.

Stalls, V., Lindenberger, J., Gobeil, S.M.-C., Henderson, R., Parks, R., Barr, M., Deyton, M., Martin, M., Janowska, K., Huang, X., et al. (2022). Cryo-EM structures of SARS-CoV-2 Omicron BA.2 spike. Cell Rep. *39*, 111009. https://doi.org/10.1016/j.celrep.2022.111009.

Sun, S., Gu, H., Cao, L., Chen, Q., Ye, Q., Yang, G., Li, R.-T., Fan, H., Deng, Y.-Q., Song, X., et al. (2021). Characterization and structural basis of a lethal mouse-adapted SARS-CoV-2. Nat. Commun. *12*, 5654. https://doi.org/10.1038/s41467-021-25903-x.

Suzuki, R., Yamasoba, D., Kimura, I., Wang, L., Kishimoto, M., Ito, J., Morioka, Y., Nao, N., Nasser, H., Uriu, K., et al. (2022). Attenuated fusogenicity and pathogenicity of SARS-CoV-2 Omicron variant. Nature *603*, 700–705. https://doi.org/10.1038/s41586-022-04462-1.

Sztain, T., Ahn, S.-H., Bogetti, A.T., Casalino, L., Goldsmith, J.A., Seitz, E., McCool, R.S., Kearns, F.L., Acosta-Reyes, F., Maji, S., et al. (2021). A glycan gate controls opening of the SARS-CoV-2 spike protein. Nat. Chem. *13*, 963–968. https://doi.org/10.1038/s41557-021-00758-3.

Tao, K., Tzou, P.L., Nouhin, J., Gupta, R.K., de Oliveira, T., Kosakovsky Pond, S.L., Fera, D., and Shafer, R.W. (2021). The biological and clinical significance of emerging SARS-CoV-2 variants. Nat. Rev. Genet. *22*, 757–773. https://doi.org/10.1038/s41576-021-00408-x.

Tian, F., Tong, B., Sun, L., Shi, S., Zheng, B., Wang, Z., Dong, X., and Zheng, P. (2021). N501Y mutation of spike protein in SARS-CoV-2 strengthens its binding to receptor ACE2. ELife *10*, e69091. https://doi.org/10.7554/eLife.69091.

van Duin, A.C.T., Dasgupta, S., Lorant, F., and Goddard, W.A., III. (2001). ReaxFF: A reactive force field for hydrocarbons. J. Phys. Chem. A *105*, 9396–9409. https://doi.org/10.1021/JP004368U.

VanBlargan, L.A., Errico, J.M., Halfmann, P., Zost, S.J., Crowe, J.E., Purcell, L.A., Kawaoka, Y., Corti, D., Fremont, D.H., and Diamond, M. (2021). An infectious SARS-CoV-2 B.1.1.529 Omicron virus escapes neutralization by several therapeutic monoclonal antibodies. Nat. Med. *28*, 490–495.

Walls, A.C., Park, Y.-J., Tortorici, M.A., Wall, A., McGuire, A.T., and Veesler, D. (2020). Structure, function, and antigenicity of the SARS-CoV-2 spike glycoprotein. Cell *181*, 281–292.e6. https://doi.org/10.1016/j.cell.2020.02.058.

Wei, C., Shan, K.-J., Wang, W., Zhang, S., Huan, Q., and Qian, W. (2021). Evidence for a mouse origin of the SARS-CoV-2 Omicron variant. J. Genet. Genomics *48*, 1111–1121.

Wilhelm, A., Widera, M., Grikscheit, K., Toptan, T., Schenk, B., Pallas, C., Metzler, M., Kohmer, N., Hoehl, S., Helfritz, F.A., et al. (2021). Reduced neutralization of SARS-CoV-2 omicron variant by vaccine sera and monoclonal antibodies. Preprint at medRxiv. https://doi.org/10.1101/2021.12.07.21267432.

Wolter, N., Jassat, W., Walaza, S., Welch, R., Moultrie, H., Groome, M., Amoako, D.G., Everatt, J., Bhiman, J.N., Scheepers, C., et al. (2022). Early assessment of the clinical severity of the SARS-CoV-2 omicron variant in South Africa: a data linkage study. Lancet *399*, 437–446. https://doi.org/10.1016/S0140-6736(22)00017-4.

Wrapp, D., Wang, N., Corbett, K.S., Goldsmith, J.A., Hsieh, C.-L., Abiona, O., Graham, B.S., and McLellan, J.S. (2020). Cryo-EM structure of the 2019-nCoV

spike in the prefusion conformation. Science *367*, 1260–1263. https://doi.org/10.1126/science.abb2507.

Wrobel, A.G., Benton, D.J., Roustan, C., Borg, A., Hussain, S., Martin, S.R., Rosenthal, P.B., Skehel, J.J., and Gamblin, S.J. (2022). Evolution of the SARS-CoV-2 spike protein in the human host. Nat. Commun. *13*, 1178. https://doi.org/10.1038/s41467-022-28768-w.

Xia, S., Chan, J.F.-W., Wang, L., Jiao, F., Chik, K.K.-H., Chu, H., Lan, Q., Xu, W., Wang, Q., Wang, C., et al. (2022). Peptide-based pan-CoV fusion inhibitors maintain high potency against SARS-CoV-2 Omicron variant. Cell Res. *32*, 404–406. https://doi.org/10.1038/s41422-022-00617-x.

Yamasoba, D., Kimura, I., Nasser, H., Morioka, Y., Nao, N., Ito, J., Uriu, K., Tsuda, M., Zahradnik, J., Shirakawa, K., et al. (2022). Virological characteristics of SARS-CoV-2 BA.2 variant. Preprint at bioRxiv. https://doi.org/10.1101/2022.02.14.480335.

Yang, K., Wang, C., White, K.I., Pfuetzner, R.A., Esquivies, L., and Brunger, A.T. (2022). Structural conservation among variants of the SARS-CoV-2 spike postfusion bundle. Proc. Natl. Acad. Sci. USA *119*, e2119467119. https://doi.org/10.1073/pnas.2119467119.

Yu, J., Li, Z., He, X., Gebre, M.S., Bondzie, E.A., Wan, H., Jacob-Dolan, C., Martinez, D.R., Nkolola, J.P., Baric, R.S., et al. (2021). Deletion of the SARS-CoV-2 spike cytoplasmic tail increases infectivity in Pseudovirus neutralization assays. J. Virol. *95*. e00044–21. https://doi.org/10.1128/JVI.00044-21.

Yurkovetskiy, L., Wang, X., Pascal, K.E., Tomkins-Tinch, C., Nyalile, T.P., Wang, Y., Baum, A., Diehl, W.E., Dauphin, A., Carbone, C., et al. (2020). Structural and functional analysis of the D614G SARS-CoV-2 spike protein variant. Cell *183*, 739–751.e8. https://doi.org/10.1016/j.cell.2020.09.032.

Zech, F., Schniertshauer, D., Jung, C., Herrmann, A., Cordsmeier, A., Xie, Q., Nchioua, R., Prelli Bozzo, C., Volcic, M., Koepke, L., et al. (2021). Spike residue 403 affects binding of coronavirus spikes to human ACE2. Nat. Commun. *12*, 6855. https://doi.org/10.1038/s41467-021-27180-0.

Zhang, J., Cai, Y., Lavine, C.L., Peng, H., Zhu, H., Anand, K., Tong, P., Gautam, A., Mayer, M.L., Rits-Volloch, S., et al. (2022a). Structural and functional impact by SARS-CoV-2 Omicron spike mutations. Cell Rep. *39*, 110729. https://doi.org/10.1016/j.celrep.2022.110729.

Zhang, L., Li, Q., Liang, Z., Li, T., Liu, S., Cui, Q., Nie, J., Wu, Q., Qu, X., Huang, W., et al. (2022b). The significant immune escape of pseudotyped SARS-CoV-2 Variant Omicron. Emerg. Microbes Infect. *11*, 1–5. https://doi.org/10.1080/22221751.2021.2017757.

Zhao, H., Lu, L., Peng, Z., Chen, L.-L., Meng, X., Zhang, C., Ip, J.D., Chan, W.-M., Chu, A.W.-H., Chan, K.-H., et al. (2022). SARS-CoV-2 Omicron variant shows less efficient replication and fusion activity when compared with delta variant in TMPRSS2-expressed cells. Emerg. Microbes Infect. *11*, 277–283. https://doi.org/10.1080/22221751.2021.2023329.

CellPress
OPEN ACCESS

STAR★METHODS

KEY RESOURCES TABLE

REAGENT or RESOURCE	SOURCE	IDENTIFIER
**Antibodies**		
Mouse monoclonal anti-V5 Spike (E9H8O)	Cell Signaling Technology	Cat#80076S; RRID: AB_2920661
Alexa Fluor 647 goat anti-mouse IgG (H+L)	Thermo Fisher	Cat#A-11004; RRID: AB_2534072
Rabbit monoclonal anti-V5 Spike	Cell Signaling Technology	Cat#13202S; RRID: AB_2687461
Mouse monoclonal anti-VSV-M (23H12)	Absolute Antibody	Cat#Ab01404-2.0
Mouse monoclonal anti-beta Actin	Abcam	Cat#ab8226; RRID: AB_306371
IRDye 800CW Goat anti-Mouse IgG (H + L)	LI-COR	Cat#926-32210; RRID: AB_621842
IRDye 680CW Goat anti-Rabbit IgG (H + L)	LI-COR	Cat#925-68071; RRID: AB_2721181
Bamlanivimab	Lilly Pharma	LY-CoV555 700 mg; Lot#D336907A
Imdevimab	Roche	REGN10897 1332 mg; Lot#N7534
Casivirimab	Roche	REGN10933 1332 mg; Lot#N7533
**Bacterial and virus strains**		
NEB® 5-alpha Competent E. coli (High Efficiency)	New England BioLabs	Cat#C2987H
XL2-Blue MRF' TM Ultracompetent cells	Agilent Technologies	Cat#200151
VSVΔG(GFP)*VSV-G	Prof. Karl-Klaus Conzelmann, Institute of Virology, LMU Munich, Germany	N/A
**Biological samples**		
Human sera	This study	N/A
**Chemicals, peptides, and recombinant proteins**		
DAPI	Sigma-Aldrich	Cat#D9542-1MG; CAS: 28718-90-3
L-Glutamine	PANBiotech	Cat#P04-80100
Penicillin-Streptomycin	PANBiotech	Cat#P06-07100
Complete ULTRA inhibitor cocktail tablet	Roche	Cat#05892791001
2-Mercaptoethanol	Sigma-Aldrich	Cat#M6250-100ML
Polyethyleneimine (PEI)	Sigma-Aldrich	Cat#408727-100ML
4 % Paraformaldehyde (PFA)	ChemCruz	Cat#sc-281692
4X Protein Sample Loading Buffer	LI-COR	Cat#928-40004
Glycerol	Sigma-Aldrich	Cat#G5516-500ML
Mowiol 4-88	ROTH	Cat#0713.1
Tween 20	Sigma-Aldrich	Cat#P7949-500ML
Tris-Cl	Sigma-Aldrich	Cat#T5941-500G
DABCO (1,4-diazabicyclo-[2,2,2]-octane)	ROTH	Cat#0718.1
HEPES	Sigma-Aldrich	Cat#H3375-250G
NaCl	Sigma-Aldrich	Cat#106404
Triton X-100	Sigma-Aldrich	Cat#T9284-100ML
Ethylenediaminetetraacetic acid (EDTA)	Sigma-Aldrich	Cat#EDS-100G
Trypsin/EDTA 0.05 % / 0.02 %	PANBiotech	Cat#P10-023100
Dulbecco's Phosphate Buffered Saline (PBS)	Thermo Fisher	Cat#14190094
Poly-L-Lysine	Sigma-Aldrich	Cat#P6282-5MG
Fetal Bovine Serum	Thermo Fisher	Cat#10270106
0.5 % Trypsin-EDTA	Thermo Fisher	Cat#15400054
Blocker Casein in PBS	Thermo Fisher	Cat#37528
Phire Hot Start II DNA-Polymerase	Thermo Fisher	Cat#F122S
dTTP (10 mM)	Invitrogen	Cat#18255018
dATP (10 mM)	Invitrogen	Cat#18252015

(*Continued on next page*)

Continued		
REAGENT or RESOURCE	SOURCE	IDENTIFIER
dCTP (10 mM)	Invitrogen	Cat#18253013
dGTP (10 mM)	Invitrogen	Cat#18254011
NEBuilder® HiFi DNA Assembly Master Mix	New England BioLabs	Cat#E2621L
Critical commercial assays		
Q5 Site-Directed Mutagenesis Kit	New England BioLabs	Cat#E0554
COVID-19 Spike-ACE2 Binding Assay Kit	RayBiotech	Cat#QHD43416
Deposited data		
Raw and analyzed data	This study	Available through Mendeley Data (https://doi.org/10.17632/jghjcrktwp.1) https://data.mendeley.com/datasets/jghjcrktwp/draft?a=8a6fe66c-bcbb-4f57-98de-5f1abed8e8d8
Experimental models: Cell lines		
Human: HEK293T cells	ATCC	CRL-3216; RRID: CVCL_0063
Human: CaCo-2 cells	ATCC	HTB-37; RRID: CVCL_0025
Mouse: I1 Hybridoma cells	ATCC	CRL-2700; RRID: CVCL_G654
Oligonucleotides		
Primers for site-directed mutagenesis of pCG_SARS-CoV-2-Hu-1-Spike C-V5-IRES_eGFP, see Table S3	This paper	N/A
Primers for site-directed mutagenesis of pCG_SARS-CoV-2-BA.1-Spike C-V5-IRES_eGFP Fw: gcttcagCACCTTCAAGTGCTACGG	Biomers.net	LPF/SSS_Fw
Primers for site-directed mutagenesis of pCG_SARS-CoV-2-BA.1-Spike C-V5-IRES_eGFP Rev: ttgcggaGTTGTACAGCACGGAGTAG	Biomers.net	LPF/SSS_Rev
Recombinant DNA		
Plasmid: pCG_SARS-CoV-2-Hu-1-Spike C-V5-IRES_eGFP (YP_009724390.1)	This study	N/A
Plasmid: pCG_SARS-CoV-2- B_1_617_2-Spike C-V5-IRES_eGFP	This study	N/A
Plasmid: pCG_SARS-CoV-2-BA.1-Spike C-V5-IRES_eGFP	This study	N/A
Plasmid: pCG_SARS-CoV-2-BA.-Spike C-V5-IRES_eGFP	This study	N/A
Plasmid: pCG_ACE2_IRES_eGFP	This study	N/A
Software and algorithms		
GraphPad Prism Version 9.2	GraphPad Software, Inc.	https://www.graphpad.com RRID: SCR_002798
LI-COR Image Studio Version 5.2	LI-COR	www.licor.com/ RRID: SCR_015795
CorelDRAW 2021 (64-Bit)	Corel Corporation	www.coreldraw.com/ RRID: SCR_014235
BioTek Gen5 3.04	Agilent Technologies	www.biotek.com
Fiji 1.53	National Institutes of Health (NIH)	imagej.nih.gov/ij/ RRID: SCR_003070
Amsterdam Modeling Suite 2020	Software for Chemistry & Materials BV	www.scm.com
Visual Molecular Dynamics 1.9.3	NIH Center for Macromolecular Modeling & Bioinformatics	www.ks.uiuc.edu/
ZEN (black edition)	Carl Zeiss Microscopy GmbH	www.micro-shop.zeiss.com RRID: SCR_013672
Other		
Dulbecco's Modified Eagle Medium	Thermo Fisher	Cat#41965039
Roswell Park Memorial Institute Medium 1640	Thermo Fisher	Cat#21875034
MEM Non-essential amino acids	Thermo Fisher	Cat#11140035

(*Continued on next page*)

CellPress
OPEN ACCESS

Continued

REAGENT or RESOURCE	SOURCE	IDENTIFIER
Opti-MEM Reduced Serum Media	Thermo Fisher	Cat#31985047
Saccharose	Sigma-Aldrich	Cat#S0389-500G
NuPAGE 4-12% Bis-Tris Gels	Invitrogen	Cat#NP0323BOX
Immobilon-FL PVDF membrane	Sigma-Aldrich	Cat#IPFL00010
Borosilicate Cover Slips, 13 mm	VWR	Cat#6310150
XbaI restriction enzyme	New England BioLabs	Cat#R0145
MluI restriction enzyme	New England BioLabs	Cat#R0198

RESOURCE AVAILABILITY

Lead contact

Further information and requests for resources and reagents should be directed to and will be fulfilled by the lead contact, Frank Kirchhoff (frank.kirchhoff@uni-ulm.de)

Materials availability

All unique reagents generated in this study are listed in the key resources table and available from the lead contact.

Data and code availability

- Original, unprocessed data are available through Mendeley Data (https://doi.org/10.17632/jghjcrktwp.1) at: https://data.mendeley.com/datasets/jghjcrktwp/draft?a=8a6fe66c-bcbb-4f57-98de-5f1abed8e8d8
- This paper does not report original code.
- Any additional information required to reanalyze the data reported in this work paper is available from the lead contact upon request.

EXPERIMENTAL MODEL AND SUBJECT DETAILS

Cell culture

All cells were cultured at 37 °C and 5% CO_2 in a humified atmosphere. HEK293T (human embryonic kidney) cells (ATCC: #CRL3216) were maintained in Dulbecco's Modified Eagle Medium supplemented with 10% (v/v) heat-inactivated fetal bovine serum, 2 mM L-glutamine, 100 μg/ml streptomycin and 100 U/ml penicillin. Caco-2 (human epithelial colorectal adenocarcinoma) cells (ATCC: #HTB-37) were cultivated in DMEM containing 20% FBS, 2mM glutamine, 100 μg/ml streptomycin and 100 U/ml penicillin, 1mM NEAA supplement. Mouse I1-Hybridoma cells (ATCC: #CRL2700) were cultured in Roswell Park Memorial Institute (RPMI) 1640 medium supplemented with 10% (v/v) heat-inactivated fetal bovine serum, 2 mM L-glutamine, 100 μg/ml streptomycin and 100 U/ml penicillin.

Sera from vaccinated individuals

Blood samples of fully BNT162b2 vaccinated individuals (n=5, three females, two males, age range 27-61 years, average age 42.2 years) were obtained after the participants information and written consent. Samples were collected 13−30 days after the second vaccination using S-Monovette Serum Gel tubes (Sarstedt). Before use, the serum was heat-treated at 56 °C for 30 min. Ulm University Medical Center Employees who were vaccinated twice, had no indication of previous SARS-CoV-2 infection and expressed interest in participating were included the present study. There were no further inclusion/exclusion parameters. Ethics approval was provided by the Ethic Committee of Ulm University (vote 99/21– FSt/Sta).

METHOD DETAILS

Expression constructs

pCG_SARS-CoV-2-Spike encoding the spike protein of SARS-CoV-2 isolate Wuhan-Hu-1(NCBI reference Sequence YP_009724390.1), pCG1_SARS-2-S-Δ18 (BA.1) and pCG1_SARS-2-SΔ18 (BA.2) were kindly provided by Stefan Pöhlmann (DPZ Göttingen, Germany). pcDNA3_1 SARS-CoV-2 S d19 B_1_617_2 was kindly provided by Beatrice H. Hahn (University of Pennsylvania). The Spike sequence of all constructs was PCR amplified and subcloned into a pCG-IRESeGFP expression construct by Gibson Assembly repairing the C-terminal deletion and introducing the V5 epitope tag. The SARS-CoV-2 S mutant plasmids were generated using Q5 Site-Directed Mutagenesis Kit (NEB #E0554). ACE2 was synthezised by Twist bioscience, PCR amplified, and subcloned into a pCG-IRES_eGFP expression construct using the restriction enzymes XbaI+MluI. All constructs were verified by sequence analysis using a commercial sequencing service (Eurofins Genomics).

Molecular dynamics simulation

Initial atomic positions of ACE2-bound to SARS-CoV-2 spike (7KNB, https://www.rcsb.org/structure/7KNB) respectably the post-fusion structure of SARS-CoV-2 spike glycoprotein (PDB id 6M3W https://www.rcsb.org/structure/6m3w) were obtained from the Protein Data Bank (Bernstein et al., 1977). Equilibrations (300 K for 0.5 ns) were performed by ReaxFF (reactive molecular dynamic) simulations (van Duin et al., 2001) within the Amsterdam Modelling Suite 2020 (http://www.scm.com). Based on the equilibrated structures, amino acids from the Wuhan-1 spike protein were replaced with the respective amino acids from Omicron BA.1 and BA.2 spike protein. These modified structures were additionally equilibrated (300 K for 0.5 ns) ReaxFF (reactive molecular dynamic) within an NVT while coupling the system to a Berendsen heat bath (T = 300 K with a coupling constant of 100 fs). The interaction energies were obtained by averaging over the last 0.1 ns of these simulations. The Visual Molecular Dynamics program (VMD 1.9.3) (Humphrey et al., 1996) was used for all visualizations.

Pseudoparticle production

To produce pseudotyped VSV(GFP)ΔG particles, HEK293T cells were transfected with Spike-expressing vectors using polyethyleneimine (PEI 1 mg/ml in H_2O). Twenty-four hours post-transfection, the cells were infected with VSVΔG(GFP)*VSV-G at a MOI of 3. The inoculum was removed 1hour post-infection. Pseudotyped VSVΔG(GFP) particles were harvested 16 h post-infection. Remaining cell debris were removed by centrifugation (500 × g for 5 min). Residual particles carrying VSV-G were blocked by adding 10% (v/v) of I1-Hybridoma supernatant (I1, mouse hybridoma supernatant from CRL-2700; ATCC) to the cell culture supernatant.

Infection assay

Caco-2 cells were infected with 100 µl of VSVΔG(GFP) pseudo particles in 96 well format. GFP-positive cells were automatically counted using a Cytation 3 microplate reader (BioTek Instruments).

Pseudoparticle inhibition

50 µl of VSVΔG(GFP) pseudo particles were preincubated for 30 min at RT with the indicated amounts of monoclonal antibodies (Bamlanivimab (LY-CoV555), Imdevimab (REGN10897), Casivirimab (REGN10933)) or sera from fully BNT162b2 vaccinated individuals and transduced on CaCo-2 cells in 96 well format. 24 hours after infection, GFP-positive cells were automatically counted by a Cytation 3 microplate reader (BioTek Instruments).

Whole-cell and cell-free lysates

To prepare whole-cell lysates, cells were collected and washed in phosphate-buffered Saline (PBS), pelleted and lysed in transmembrane lysis buffer, containing protease inhibitor (1:500). After 5 min of incubation on ice, supernatants were cleared by centrifugation (4 °C, 20 min, 20,817 × g). To prepare WB lysates of viral particles, the supernatants were layered on a cushion of 20% sucrose and centrifuged (4 °C, 90 min, 20,817 × g). The virus pellet was lysed in transmembrane lysis buffer, mixed with 4x Protein Sample Loading Buffer (LI-COR) containing 10% β-mercaptoethanol (Sigma Aldrich) and denaturized at 95 °C for 10 min.

SDS-PAGE and immunoblotting

SDS-PAGE and immunoblotting was performed as previously described (Zech et al., 2021). In brief, whole cell lysates were mixed with 4x Protein Sample Loading Buffer (LI-COR) containing 10% β-mercaptoethanol (Sigma Aldrich), heated at 95 °C for 20 min, separated on NuPAGE 4-12% Bis-Tris Gels (Invitrogen) for 90 min at 120 V and blotted at constant 30 V for 30 min onto Immobilon-FL PVDF membrane. After the transfer, the membrane was blocked in 1% Casein in PBS. Proteins were stained using primary antibodies directed against rabbit anti-V5 (Cell Signaling #13202; 1:1000), VSV-M (Absolute Antibody, 23H12, #Ab01404-2.0; 1:2000), actin (Anti-beta Actin antibody Abcam, ab8226, 1:5000,) and Infrared Dye labeled secondary antibodies (LI-COR IRDye) IRDye 800CW Goat anti-Mouse #926-32210, IRDye 680CW Goat anti-Rabbit (#925-68071), all 1:20,000. Proteins were detected using a LI-COR Odyssey scanner and band intensities were quantified using LI-COR Image Studio version 5.

ACE2 interaction assay

HEK293T cells expressing Spike were collected 48 h after the transfection, washed with phosphate-buffered saline (PBS), lysed in a non-denaturizing lysis buffer. Interaction between Spike protein and ACE2 was assessed through a Spike-ACE2 binding assay kit (COVID-19 Spike-ACE2 binding assay II, Ray Bio). Briefly, 10 µl of WCLs were diluted 1:5 in 1x assay diluent buffer (RayBio), added to ACE2 coated wells (RayBio) and incubated for 2 h with shaking. After washing extensively with the provided wash buffer (RayBio, #EL-ITEMB), the wells were incubated 1 h with 100 µl anti-V5(Mouse) (1:1,000, Cell Signalling, #80076), washed and incubated for 1 h with 100 µl anti-mouse-HRP (1:1,000, RayBio). After washing, the samples were incubated with 50 µl of TMB Substrate Solution (RayBio, #EL-TMB) for 30 min. The reaction was stopped by the addition of 50 µl Stop Solution (RayBio, #ELSTOP) and absorbance was measured at 450 nm with a baseline correction at 650 nm.

Immunofluorescence

HEK293T cells were plated in 12-well tissue culture dishes on 13-mm round borosilicate cover slips pre-coated with poly-L-lysine. 24 hours after, the cells were transfected with expression constructs for Spike protein using polyethyleneimine (PEI 1 mg/ml in H_2O). 24 hours after transfection, cells were washed with cold PBS and fixed in 4% paraformaldehyde solution (PFA) for 20 min at

RT, permeabilized and blocked in PBS containing 0.5% Triton X-100 and 5% FCS for 30 min at RT. Thereafter, samples were washed with PBS and incubated for 2 h at 4°C with primary antibody (anti-V5(Mouse) (1:1,000, Cell Signalling, #80076S)) diluted in PBS. The samples were washed with PBS/0.1% Tween 20 and incubated in the dark for 2 h at 4°C with the secondary antibody (Alexa Fluor-647-conjugated anti-mouse antibody, 1:1000, Thermo Fisher Scientific) and 500 ng/ml DAPI. After washing with PBS-T and water, cover slips were mounted on microscopy slides. Images were acquired using a Zeiss LSM800 confocal laser scanning microscope with ZEN imaging software (Zeiss).

Quantification of syncytia formation

To detect formation of syncytia, HEK293T cells were co-transfected with ACE2 and Spike expressing vectors using polyethyleneimine (PEI 1 mg/ml in H_2O). Twenty-four hours post-transfection, fluorescence microscopy images were acquired using the Cytation 3 microplate reader (BioTek Instruments) and the GFP area was quantified using ImageJ.

NGS sequence analysis

CovSpectrum (Open) (Chen et al., 2021) was used to identify genomes on GenBank that were assigned as Omicron (BA*) but purported to have an S at position 371. 100 Biosamples associated with these samples were randomly selected for analysis. For these, we queried the ENA for raw sequencing reads, identifying 58 genomes with raw reads available. For each we mapped fastq files to the Hu-1 reference genome using minimap2 and made coverage plots for a region including S:371-375.

QUANTIFICATION AND STATISTICAL ANALYSIS

Spike-mediated infection is presented as the mean of three biological replicates, each consisting of three technical replicates. Infection was normalized to the SARS-CoV-2 Spike Wuhan-1 (Hu-1) sample. Immunoblot analysis was performed in biological triplicates with one representative blot shown. Band intensity is represented by mean values of three biological replicates. Linear correlations and statistics were performed using GraphPad PRISM 9.2 simple linear regression. Syncytia formation is presented as the mean of three biological replicates, of which one representative image is shown. Spike/ACE2 binding is presented as the mean of four biological replicates, each consisting of three technical replicates. Binding was normalized to the value obtained for the SARS-CoV-2 Spike Wuhan-1 (Hu-1) sample. Serum neutralization is presented as the mean neutralization efficiency by sera derived from five individual donors each performed in two technical replicates. Neutralization was normalized to the SARS-CoV-2 Spike Wuhan-1 (Hu-1) sample. Neutralization by monoclonal antibodies is presented as mean from three biological replicates, each consisting of three technical replicates. $TCID_{50}$ values of Serum neutralization and IC_{50} values of antibody inhibition were calculated by a non-linear regression model with variable slope. Error bars show the standard error of means (SEM).

Statistical analyses were performed using GraphPad PRISM 9.2 (GraphPad Software). Statistical significance was analysed by two-tailed Student's t-test with Welch's correction (correlation data of Spike expression/processing and infection rate, cell-cell fusion assay) or One-Way ANOVA with multiple comparison against the Wuhan-Hu-1 values (Spike-mediated infection, ACE-2 interaction assay). Statistical parameters are stated in the figure legends. No methods were used to determine whether the data met assumptions of the statistical approach. Significant differences are indicated as *$p < 0.05$; **$p < 0.01$; ***$p < 0.001$.

Cell Host & Microbe, Volume *30*

Supplemental information

Determinants of Spike infectivity, processing, and neutralization in SARS-CoV-2 Omicron subvariants BA.1 and BA.2

Chiara Pastorio, Fabian Zech, Sabrina Noettger, Christoph Jung, Timo Jacob, Theo Sanderson, Konstantin M.J. Sparrer, and Frank Kirchhoff

SUPPLEMENTAL FIGURES

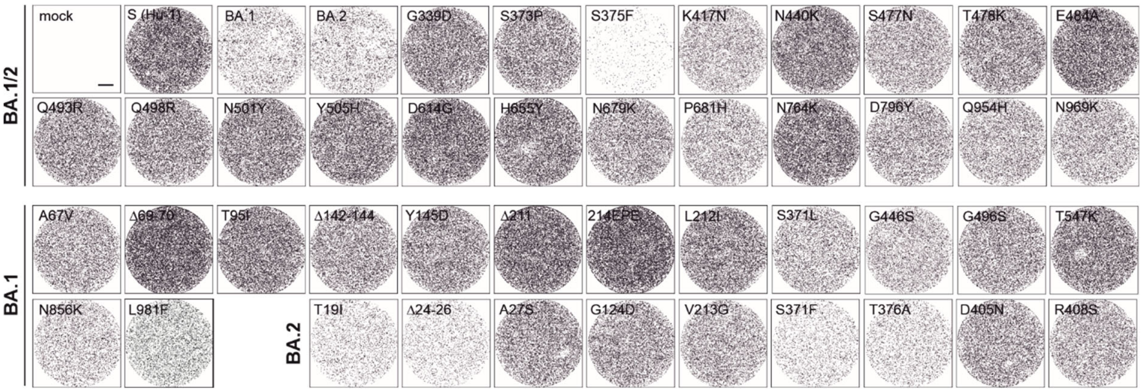

Figure S1 (related to Figure 2). Infection of CaCo-2 cells by VSVpp containing WT or mutant S proteins.

Images of CaCo-2 cells transduced with VSVΔG-GFP pseudotyped with the Hu-1 or mutant SARS-CoV-2 S proteins. Successful infection events (GFP+ cells) are displayed as black dots. Scale bar, 1.5 μm.

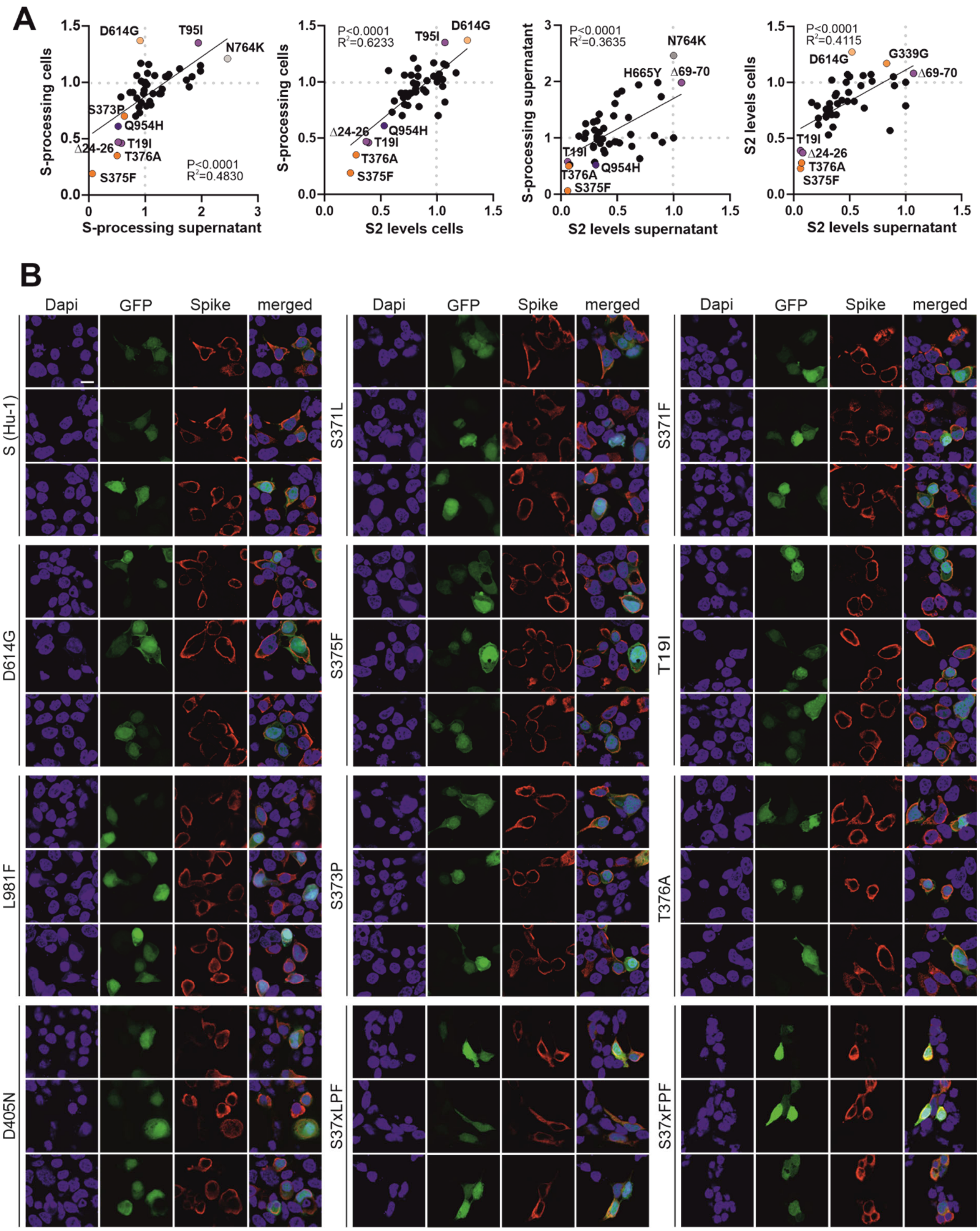

Figure S2 (related to Figure 3). Correlation analyses and localization of S proteins.

(A) Correlation of the between the indicated parameters. S2 expression levels and S/S2 processing of mutant S proteins were normalized to the parental Hu-1 S (set to 1).

(B) Immunofluorescence images of HEK293T cells expressing the parental Hu-1 or indicated mutant S proteins. Scale bar, 10 µm.

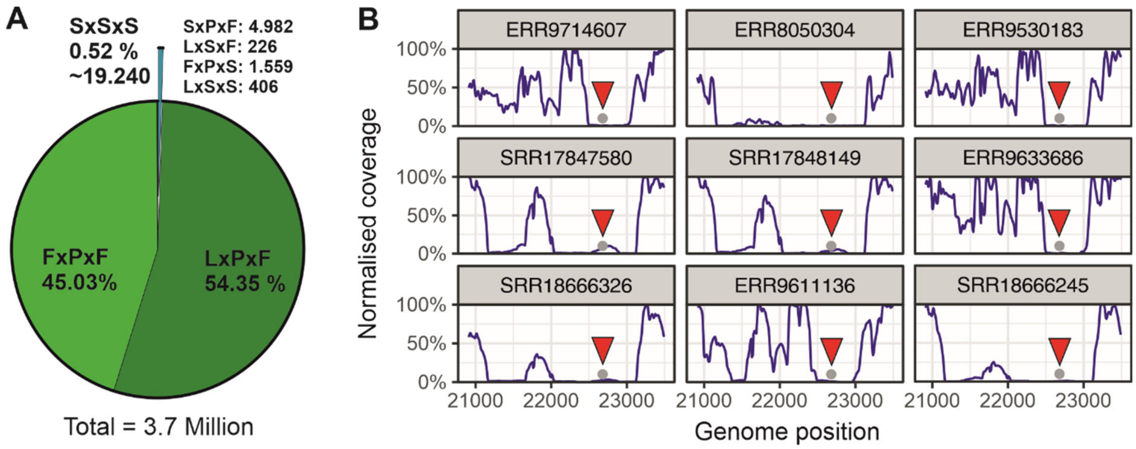

Figure S3 (related to Figure 4). Apparent changes to serine residues at amino acid positions 371, 373 and 375 of Omicron Spike. (A) Pie chart depicting the frequency of GSAID deposited sequences with indicated Spike mutations, retrieved from CoVspectrum on 23.06.2022. (B) Normalised coverage plots for randomly selected Omicron genomes with consensus sequences having a "revertant" S:371S show drops of coverage at this position. The red arrow indicates the S:371-5 location.

SUPPLEMENTAL TABLES

Table S1 (related to Figure 5). Impact of mutations in Omicron BA.1 and BA.2 Spike on infection, processing, ACE2 binding, syncytia formation and neutralization.

Residue	Infection	Processing				ACE2 interaction	Syncytia formation	Serum neutralization
		S Sups	S2 Sups	S Cells	S2 Cells			
Hu-1	1.00±0.00	0.60±0.06	0.40±0.06	0.59±0.05	0.41±0.05	1.01±0.01	1.00±0.00	1.00±0.00
Delta	1.06±0.05	n.d.	n.d.	n.d.	n.d.	n.d.	1.11±0.04	6.07±2.99
BA.1	0.41±0.05	0.53±0.02	0.47±0.02	0.66±0.03	0.34±0.03	1.14±0.06	0.67±0.02	16.64±2.91
BA.2	0.26±0.05	0.60±0.03	0.40±0.03	0.65±0.03	0.35±0.03	0.82±0.05	0.69±0.03	16.30±5.95
G339D	1.10±0.02	0.58±0.06	0.42±0.06	0.57±0.06	0.43±0.06	n.d.	1.16±0.09	2.77±1.06
S373P	0.81±0.14	0.60±0.08	0.40±0.08	0.67±0.07	0.33±0.07	0.97±0.04	1.06±0.11	4.43±1.50
S375F	0.10±0.00	0.92±0.04	0.08±0.04	0.87±0.07	0.13±0.07	0.54±0.07	0.15±0.00	1.45±0.77
K417N	1.06±0.02	0.54±0.07	0.46±0.07	0.64±0.06	0.36±0.06	n.d.	1.23±0.02	4.38±1.44
N440K	1.05±0.09	0.58±0.08	0.42±0.08	0.61±0.06	0.39±0.06	n.d.	1.30±0.08	3.94±1.29
S477N	0.99±0.06	0.60±0.08	0.40±0.08	0.65±0.05	0.35±0.05	n.d.	1.19±0.01	2.23±0.67
T478K	1.04±0.07	0.58±0.11	0.42±0.11	0.61±0.05	0.39±0.05	n.d.	1.21±0.01	2.18±1.39
E484A	0.82±0.10	0.51±0.15	0.49±0.15	0.61±0.05	0.39±0.05	n.d.	1.27±0.06	1.38±0.59
Q493R	1.02±0.03	0.60±0.05	0.40±0.05	0.59±0.06	0.41±0.06	n.d.	1.20±0.06	3.63±1.22
Q498R	1.03±0.08	0.64±0.05	0.36±0.05	0.63±0.08	0.37±0.08	n.d.	1.21±0.05	2.36±0.72
N501Y	0.98±0.07	0.68±0.06	0.32±0.06	0.61±0.08	0.39±0.08	1.22±0.07	1.10±0.10	3.39±1.19
Y505H	0.93±0.08	0.48±0.12	0.52±0.12	0.58±0.05	0.42±0.05	n.d.	1.20±0.05	2.94±1.02
D614G	1.20±0.07	0.65±0.07	0.35±0.07	0.52±0.05	0.48±0.05	0.81±0.08	1.36±0.04	2.58±0.79
H655Y	0.95±0.09	0.44±0.09	0.56±0.09	0.57±0.04	0.43±0.04	n.d.	1.18±0.05	1.47±0.36
N679K	0.73±0.15	0.57±0.07	0.43±0.07	0.56±0.07	0.44±0.07	n.d.	1.06±0.05	2.62±0.88
P681H	0.85±0.05	0.59±0.05	0.41±0.05	0.58±0.06	0.42±0.06	n.d.	1.18±0.02	2.37±0.65
N764K	1.12±0.06	0.34±0.07	0.66±0.07	0.55±0.04	0.45±0.04	n.d.	1.26±0.06	2.44±0.84
D796Y	0.82±0.08	0.68±0.04	0.32±0.04	0.63±0.09	0.37±0.09	n.d.	1.10±0.04	0.81±0.31
Q954H	0.70±0.01	0.73±0.07	0.27±0.07	0.70±0.08	0.30±0.08	n.d.	0.91±0.04	0.76±0.19
N969K	0.58±0.05	0.54±0.10	0.46±0.10	0.62±0.05	0.38±0.05	n.d.	0.79±0.02	1.23±0.31
A67V	0.75±0.03	0.56±0.05	0.44±0.05	0.61±0.04	0.39±0.04	n.d.	1.04±0.02	0.23±0.09
Δ69-70	1.11±0.01	0.45±0.06	0.55±0.06	0.57±0.03	0.43±0.03	n.d.	1.29±0.04	0.52±0.13
T95I	1.13±0.03	0.44±0.07	0.56±0.07	0.52±0.05	0.48±0.05	n.d.	1.29±0.04	2.52±1.02
Δ142-144	0.86±0.02	0.49±0.11	0.51±0.11	0.58±0.07	0.42±0.07	n.d.	1.05±0.04	4.22±1.87
Y145D	0.80±0.04	0.46±0.08	0.54±0.08	0.60±0.06	0.40±0.06	n.d.	1.09±0.05	6.98±2.45
Δ211	0.76±0.03	0.55±0.12	0.45±0.12	0.64±0.06	0.36±0.06	n.d.	1.09±0.06	0.08±0.02
214EPE	1.04±0.04	0.48±0.07	0.52±0.07	0.60±0.02	0.40±0.02	n.d.	1.18±0.02	6.95±2.09
L212I	1.13±0.02	0.65±0.06	0.35±0.06	0.56±0.04	0.44±0.04	n.d.	1.28±0.04	5.30±1.54
S371L	0.36±0.05	0.77±0.10	0.23±0.10	0.65±0.06	0.35±0.06	0.74±0.07	0.62±0.02	3.07±1.03
G446S	0.83±0.14	0.62±0.11	0.38±0.11	0.66±0.06	0.34±0.06	n.d.	1.19±0.05	1.25±0.39
G496S	1.00±0.05	0.57±0.08	0.43±0.08	0.58±0.06	0.42±0.06	n.d.	1.27±0.03	3.06±0.91
T547K	0.86±0.03	0.69±0.11	0.31±0.11	0.59±0.05	0.41±0.05	n.d.	1.14±0.04	1.42±0.35
N856K	0.44±0.09	0.54±0.09	0.46±0.09	0.57±0.03	0.43±0.03	0.82±0.13	0.99±0.11	1.18±0.40
L981F	1.20±0.08	0.60±0.17	0.40±0.17	0.68±0.03	0.32±0.03	0.80±0.17	1.50±0.04	0.96±0.41
T19I	0.53±0.07	0.59±0.25	0.41±0.25	0.76±0.06	0.24±0.06	n.d.	0.62±0.03	1.25±0.43
Δ24-26	0.66±0.04	0.61±0.20	0.39±0.20	0.76±0.05	0.24±0.05	n.d.	0.64±0.02	2.25±1.68
A27S	1.00±0.07	0.63±0.03	0.37±0.03	0.62±0.02	0.38±0.02	n.d.	1.24±0.07	5.63±2.12
G142D	0.99±0.02	0.41±0.12	0.59±0.12	0.58±0.03	0.42±0.03	n.d.	1.22±0.03	4.54±2.58
V213G	1.06±0.05	0.52±0.09	0.48±0.09	0.58±0.03	0.42±0.03	n.d.	1.18±0.07	4.35±1.72
S371F	0.50±0.01	0.75±0.08	0.25±0.08	0.61±0.02	0.39±0.02	0.91±0.10	0.15±0.00	3.93±1.77
T376A	0.44±0.05	0.76±0.11	0.24±0.11	0.80±0.06	0.20±0.06	0.58±0.11	0.20±0.01	2.09±0.69
D405N	1.08±0.07	0.66±0.08	0.34±0.08	0.65±0.03	0.35±0.03	n.d.	1.26±0.06	1.58±0.97
R408S	1.12±0.06	0.60±0.05	0.40±0.05	0.62±0.03	0.38±0.03	n.d.	1.28±0.09	5.30±1.37
SSS/LPF	0.25±0.01	n.d.	n.d.	n.d.	n.d.	0.59±0.03	0.18±0.00	n.d.
SSS/FPF	0.28±0.03	n.d.	n.d.	n.d.	n.d.	0.76±0.17	0.20±0.00	n.d.

For each mutation in Spike analyzed, the table lists: normalized PP infection of CaCo-2 cells (Infection); levels of full-length Spike in supernatants (S Sups) and cells (S Cells); levels of S2 Spike subunit in supernatants (S2 Sups) and cells (S2 Cells); binding of Spike to ACE2 (ACE2 interaction); automated quantification of syncytia formation in HEK293T cells expressing the indicated mutant S proteins and Human ACE2 (Syncytia formation); average TCID50 values obtained for neutralization of the indicated mutant S proteins by sera from five vaccinated individuals relative to those obtained for the Hu-1 S (Serum neutralization). See methods for detail.

Table S2 (related to Figure 6). Origin and neutralizing activity of sera from BNT/BNT vaccinated individuals.

Donor	AGE	Sex
1	61	w
2	28	w
3	58	m
4	27	w
5	37	m

WT

Donor	S Hu-1	Delta	BA.1	BA.2
1	0,15	0,69	3,88	0,7
2	0,9	1,52	>10	>10
3	0,4	7,11	>10	>10
4	0,99	3,36	>10	>10
5	0,29	0,82	5,44	6,03

Colors:

NTD
RBD
RBM
S1
S2
S2'

IC50 Values BA.1/BA.2

Donor	G339D	S373P	S375F	K417N	N440K	S477N	T478K	E484A	Q493R	Q498R
1	0,29	0,88	0,17	1,23	1,09	0,61	0,46	0,51	0,89	0,6
2	1,07	0,97	0,29	0,87	0,73	0,55	0,12	0,2	0,34	0,55
3	1,1	1,82	0,31	2,79	2,15	1,28	2,9	0,78	1,86	1,54
4	6,73	9,11	4,43	4,4	5,33	2,49	0,1	1,14	6,07	2,57
5	0,34	0,42	0,16	0,4	0,27	0,22	0,1	0,07	0,31	0,22

Donor	N501Y	Y505H	D614G	H655Y	N679K	P681H	N764K	D796Y	Q954H	N969K
1	0,84	0,85	0,69	0,29	0,77	0,68	0,77	0,28	0,17	0,32
2	0,41	0,43	0,58	0,43	0,41	0,62	0,41	0,19	0,15	0,33
3	2,53	1,77	1,45	0,82	1,17	0,9	1,42	0,45	0,42	0,57
4	3,63	3,35	3,23	2,16	3,8	2,88	2,02	0,63	0,95	1,45
5	0,27	0,21	0,22	0,2	0,24	0,42	0,31	0,07	0,13	0,21

IC50 Values BA.1

Donor	A67V	d69-70	T95I	d142-144	Y145D	d211	214EPE	L212I	S371L	G446S	G496S	T547K	N856K	L981F
1	0,01	0,12	0,31	0,49	1,32	0,01	1,47	1,03	0,84	0,16	0,81	0,33	0,32	0,38
2	0,26	0,27	0,79	1,09	1,01	0,06	2,22	1,04	0,64	0,28	0,74	0,39	0,21	0,24
3	0,04	0,19	2,59	4,61	5,18	0,02	2,92	3,48	1,84	1,03	1,7	0,85	0,59	0,37
4	0,55	0,84	1,41	2,64	9,56	0,03	9,89	7,56	3,91	1,55	3,77	1,49	1,83	0,72
5	0,04	0,05	0,5	0,68	0,38	0,05	0,64	0,6	0,15	0,21	0,29	0,23	0,07	0,1

IC50 Values BA.2

Donor	T19I	d24-26	A27S	G142D	V213G	S371F	T376A	D405N	R408S
1	0,16	0	1,94	0,43	0,31	0,36	0,39	0,04	1,25
2	0,22	1,08	0,96	0,92	0,93	0,21	0,5	0,44	1,96
3	0,75	0,11	1,92	5,88	2,74	2,66	1,17	0,06	2,96
4	2,51	8,79	7,39	2,18	9,74	9,24	3,88	1,64	6,73
5	0,15	0,25	0,56	0,53	0,55	0,27	0,12	1,53	0,52

monoclonal ABs IC50

	WT	21k	21l	K417N	N440K	G446S	S477N	T478K	E484A	Q493R	G496S	Q498R	N501Y	Y505H
amlanivimab	0,02667	>20	>20	n.a	0,02307	0,02157	n.a	n.a	>20	>20	0,02022	n.a	n.a	n.a
Casivirimab	0,03493	>20	>20	0,433	n.a	n.a	0,1565	0,1187	0,1395	0,08354	0,01754	0,09144	n.a	0,03691
Imdevimab	0,0985	>20	13,7	n.a	>20	>20	n.a.	n.a.	n.a.	n.a.	n.a.	0,1605	0,1655	n.a.
